# Novel insights into the multifaceted roles of m^6^A-modified LncRNAs in cancers: biological functions and therapeutic applications

**DOI:** 10.1186/s40364-023-00484-7

**Published:** 2023-04-17

**Authors:** Jinxin Tang, Jinhui Zhang, Yu Lu, Jieyu He, Hua Wang, Binfeng Liu, Chao Tu, Zhihong Li

**Affiliations:** 1 Department of Orthopaedics, The Second Xiangya Hospital of Central South University, Changsha, Hunan 410011 China; 2 Hunan Key Laboratory of Tumor Models and Individualized Medicine, The Second Xiangya Hospital of Central South University, Changsha, Hunan 410011 China; 3 Xiangya School of Medicine, Central South University, Changsha, Hunan 410011 China; 4 Department of Geriatrics, The Second Xiangya Hospital of Central South University, Changsha, Hunan 410011 China

**Keywords:** m^6^A-modification, LncRNA, Cancer, Sarcoma, Biomarker

## Abstract

N6-methyladenosine (m^6^A) is considered as the most common and important internal transcript modification in several diseases like type 2 diabetes, schizophrenia and especially cancer. As a main target of m^6^A methylation, long non-coding RNAs (lncRNAs) have been proved to regulate cellular processes at various levels, including epigenetic modification, transcriptional, post-transcriptional, translational and post-translational regulation. Recently, accumulating evidence suggests that m^6^A-modified lncRNAs greatly participate in the tumorigenesis of cancers. In this review, we systematically summarized the biogenesis of m^6^A-modified lncRNAs and the identified m^6^A-lncRNAs in a variety of cancers, as well as their potential diagnostic and therapeutic applications as biomarkers and therapeutic targets, hoping to shed light on the novel strategies for cancer treatment.

## Introduction

**Fig. 1:**
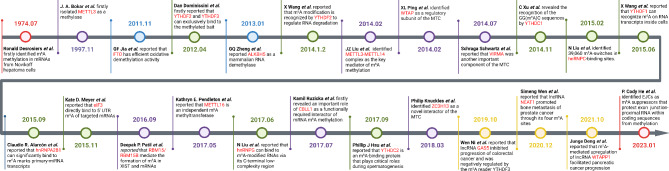
Timeline of m^6^A methylation. This figure briefly shows the important studies and time nodes during the exploration of m^6^A methylation. Red: the earlies and the latest research about m^6^A methylation; Purple: research about m^6^A writers; Blue: research about m^6^A erasers; Green: research about m^6^A readers; Yellow: research about m^6^A-modified lncRNAs.

Methylation modification is an essential component of epigenetic modification in eukaryotic cells [1]. To date, accumulating evidence has shown numerous methylation modifications that regulate RNA behaviors, including N1-methyladenosine (m^1^A), 5-methylcytosine (m^5^C), 5-hydroxymethylcytosine (hm^5^C) and N6-methyladenosine (m^6^A) [2]. Among all these modifications, m^6^A methylation is considered as the most common, abundant, and conserved internal transcript modification. In 1974, Ronald Desrosiers et al. first identified m^6^A methylation in mRNAs from Novikoff hepatoma cells [3]. After this landmark, lots of proteins that regulate m^6^A methylation were gradually identified, which have established a thorough m^6^A methylation system (Fig. 1). Most recently, P. Cody He et al. identified exon junction complexes (EJCs) as m^6^A suppressors that prevented the m^6^A methylation of exon junction-proximal RNA within coding sequences, which significantly supplemented the current understanding of m^6^A methylation progression [4]. With the deepening of the research on the role of m^6^A methylation, studies have shown a close relationship between m^6^A methylation and multiple diseases including cancer, major depressive disorder, autism spectrum disorder, schizophrenia, Alzheimer’s disease and type 2 diabetes [5]. However, the detailed mechanisms of how m^6^A methylation explicitly mediates these diseases is still under intensive investigation.

Long non-coding RNAs (lncRNAs) are generally defined as transcripts longer than 200 nucleotides (nt) in length without protein coding ability, including enhancer RNAs (eRNAs), intergenic transcripts and sense or antisense transcripts overlapping other genes [6, 7]. However, there are also researches demonstrated that some annotated lncRNAs encoded for microproteins in practice [7]. Further studies revealed that these microproteins were coded by small open reading frames (smORFs) in lncRNAs and these smORFs-derived microproteins acted key roles in the physiological and pathological activities of muscles [8]. For example, a smORF of 138 nt was identified in lncRNA long intergenic non-protein coding RNA 00948 (LINC00948), which encoded a conserved micropeptide named myoregulin (MLN). MLN could directly interact with sarcoplasmic reticulum Ca^2+^-ATPase (SERCA) to obstruct Ca^2+^ uptake into the sarcoplasmic reticulum (SR), which played an important role in skeletal muscle physiology [9]. It is now widely confirmed that lncRNAs exert significant functions in transcriptional regulation, nuclear domains organization and regulation of proteins and RNAs [10] [11]. With the deepening of the research, lncRNAs have been found to share an important role in cancer progression. A multitude of lowly expressed and often nonconserved lncRNAs were pervasively identified in transcripts of human cancer genome [12] [13] [14] [15]. Lots of studies further proved that lncRNAs were recurrently deregulated in cancers and functioned as tumor suppressors or oncogenes through sponging miRNAs [16, 17]. Moreover, mechanism researches revealed that lncRNAs acted an essential role in numerous pathways including p53 [18], STAT3 [19] and mTOR [20] cascades. Due to their prominent functions in tumorigenesis and progression, lncRNAs have attracted great attention in recent years.

With the development of MeRIP-m^6^A-seq technology, m^6^A methylations were widely identified in lncRNAs and their interaction aroused wide attention in clinical research and medical development, especially in the oncology field. Nevertheless, the potential role of m^6^A-modified lncRNAs as biomarkers for cancer detection keeps undefined. In this review, we summarized all the identified m^6^A-modified lncRNAs that participate in the cancer progression and the corresponding biological functions, aiming to provide a novel insight in cancer diagnosis and treatment.

## Process of m^6^A methylation

**Fig. 2:**
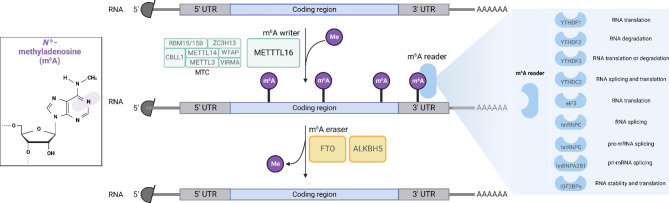
Composition of m^6^A writers, readers, erasers. This figure concisely shows the composition of well-known m^6^A writers (METTL3, METTL14, WTAP, VIRMA, RBM15/15B, ZC3H13, CBLL1 and METTL16), erasers (FTO and ALKBH5) and readers (YTHDF1, YTHDF2, YTHDF3 YTHDC1, YTHDC2, eIF3, hnRNPC, hnRNPG, hnRNPA2B1 and IGF2BP1/2/3). M6A is installed by writers, removed by erasers and recognized by readers

m^6^A modifies RNA via the dynamic interaction between numerous proteins. Generally, m^6^A is installed by methyltransferases (writers), recognized by m^6^A-binding proteins (readers) and removed by demethylases (erasers) (Fig. 2) [21].

### m^6^A writers

Thus far, researchers have found two main m^6^A writers: methyltransferase complex (MTC) and methyltransferase-like 16 (METTL16). MTC is a protein complex with molecular weight of ~ 1MDa [22], which consists of six core subunits: METTL3, METTL14, WT1 associated protein (WTAP), vir like m^6^A methyltransferase associated (VIRMA), RNA-binding motif protein 15/15B (RBM15/15B), zinc finger CCCH-type containing 13 (ZC3H13) and Cbl proto-oncogene like 1 (CBLL1) [23].

METTL3 was first found in 1997 and is now regarded as the most important catalytic subunit of MTC [24]. In 2014, METTL3-METTL14 complex was proved to the key mediator of m^6^A methylation, while WTAP was identified as a regulatory subunit of the m^6^A methyltransferase [25, 26]. Previous studies have indicated that silencing of METTL3 directly causes a decrease of m^6^A level in mammalian embryonic stem cells (ESCs), HeLa cells and HepG2 cells [27]. Meanwhile, METTL3 has been found to directly mediate the progression of some diseases, such as leukemia, as suppression of METTL3 results in a selective decrease of m^6^a levels on leukemogenic mRNAs [28]. These facts indicate that METTL3 acts a significant role in m^6^A methylation.

METTL14 is a homolog of METTL3, which acts together with METLL3 to form m^6^A methylation [29]. During m^6^A methylation process, METTL14 can shape a stable heterodimer core complex with METTL3 and synergistically increase METTL3 activities [30].

WTAP is a pivotal cofactor of METTL3-METTL14 heterodimer, as it can recruit METTL3 and METTL14 to be localized in nuclear speckles and catalyze m^6^A methylation [31].

VIRMA, which was previously known as KIAA1429, is also an important component of MTC. In 2014, Schraga Schwartz *et al*. reported that VIRMA was another important component of the methyltransferase complex [32]. It can interact with WTAP and install m^6^A to the 3’ UTR of aimed mRNA and mediate selective polyadenylation in HeLa cells [33, 34].

RBM15 and RBM15B are two paralogous RNA-binding proteins. In 2016, Deepak P. Patil et al. proved that RBM15/RBM15B mediate the formation of m^6^A in X inactive specific transcript (XIST) and mRNAs, which promotes XIST-mediated gene repression [35]. Functionally, they can interact with METTL3-METTL14 heterodimer through a WTAP-dependent pathway and recruit the methyltransferase complex to U-rich regions of targeted RNA [35].

ZC3H13, which is a zinc finger protein, plays an imperative role in the WTAP localization and m^6^A deposition. In 2018, Philip Knuckles et al. identified ZC3H13 as a novel interactor of the m^6^A methyltransferase complex, which is conserved in drosophila and mice [36]. Further studies showed that knockdown of ZC3H13 in mouse embryonic stem cell significantly decreased nuclear content of WTAP and m^6^A level on mRNA [37]. CBLL1, also known as HAKAI, is an E3 ubiquitin ligase that is a conserved component of the methyltransferase complex in drosophila and human cells.

In 2017, Kamil Ruzicka et al. first revealed an important role of CBLL1 as a functionally required interactor of mRNA m^6^A methylation in the identification of a conserved set of proteins forming the m^6^A writer complex in Arabidopsis [38]. Its ubiquitination domain is required for stabilization of MTC components and intact m^6^A deposition [39].

In addition to MTC, METTL16 is also an independent RNA methyltransferase responsible for the deposition of m^6^A in some transcripts including U6 snRNA, ncRNAs and pre-mRNAs [40, 41]. In 2017, Kathryn E. Pendleton et al. showed that METTL16 is the conserved U6 snRNA methyltransferase, which regulates SAM synthetase intron retention and is required for normal m^6^A methylation profiles [40]. It has been shown that > 90% of the METTL16-specific m^6^A targets are different with the targets of MTC [42]. Meanwhile, analyses suggested that METTL16 is the most essential gene for the survival of the vast majority of cancer cells among all the METTL family members, which indicates a potential role in cancer treatment [42].

### m^6^A readers

m^6^A readers are a group of binding proteins that can specifically bind to m^6^A marked RNAs and mediate their activity. For different downstream biological functions, there are varies of m^6^A readers that participate in the m^6^A recognition. One of the most important classes of m^6^A readers belongs to the YT521-B homology (YTH) domain family, including YTH domain family protein 1 (YTHDF1), YTHDF2, YTHDF3, YTH domain containing 1(YTHDC1) and YTHDC2 [43].

The identification of all these m^6^A readers came after the continuous effort of lots of scientists. In 2012, Dan Dominissini et al. first reported that YTHDF2 and YTHDF3 can exclusively bind to the methylated bait, and YTHDC1 was found to participate in specifying transcripts for nuclear degradation [44]. In 2014, C Xu et al. further revealed the specific mode of m^6^A-YTH binding and explained the recognition of the GG(m^6^A)C sequences by YTHDC1 [45]. In 2015, Xiao Wang et al. ulteriorly found that YTHDF1 could recognize m^6^A on RNA transcripts inside cells and mediate translation promotion to increase translation efficiency via a m^6^A-dependent manner, while YTHDF2 could mediate degradation of m^6^A-modified RNA transcripts to control their lifetime [46]. In 2017, Phillip J Hsu et al. confirmed YTHDC2 was a m^6^A reader in mammalian spermatogenesis [47].

Functionally, [43]YTHDF1 was confirmed to increase the translation efficiency of m^6^A methylated mRNAs. It can directly bind to exact GRAC sites around stop codons in mRNAs and then recruit mRNAs to ribosomes [46]. Meanwhile, YTHDF1 can also interact with initiation factors including eukaryotic translation initiation factor 3 (eIF3), eukaryotic translation initiation factor 4G (eIF4G) and G3BP stress granule assembly factor 1 (G3BP1) [46]. Collectively, YTHDF1 increases translation efficiency and ensures effective protein production from mRNAs that are marked by m^6^A. In contrast to YTHDF1, YTHDF2 was considered to destabilizes m^6^A-containing RNA. The carboxyterminal domain of YTHDF2 is responsible for the recognition of m^6^A-modified RNA, while the amino-terminal domain regulates the localization of the YTHDF2-RNA complex to cellular RNA decay sites [43]. Further studies showed that YTHDF2 could recognize m^6^A modifications in both the coding and noncoding regions of RNA via its carboxyterminal-terminal YTH domain, and then recruit CCR4-NOT complex to m^6^A methylated RNA via the interaction between the YTHDF2 amino-terminal region and the SH domain of CCR4-NOT transcription complex subunit 1 (CNOT1) subunit, thus to accelerate the deadenylation and degradation of m^6^A-methylated RNA [48]. YTHDF3 has been considered to act a synergistic role, which can cooperate with both YTHDF1 and YTHDF2 to promote the translation or degradation of targeted mRNAs [49, 50].

YTHDC1 was confirmed to modulate mRNA splice site selection in a concentration-dependent manner. It can recruit pre-mRNA splicing factor serine and arginine rich splicing factor 3 (SRSF3) and obstruct serine and arginine rich splicing factor 10 (SRSF10) mRNA binding to promote exon inclusion and regulate mRNA splicing [51]. In addition, YTHDC2 was first confirmed to be a m^6^A reader in mammalian spermatogenesis. It was showed to enhance the translation efficiency of its targets and also decreases their mRNA abundance, which plays a critical role during spermatogenesis [47]. Further study indicated that YTHDC2 is recruited to m^6^A marks within the coding regions of mRNAs to resolve mRNA secondary structures and positively regulate translation [52].

Along with the YTH family, there are also other proteins that act as m^6^A readers. EIF3 is a large multiprotein complex comprising 13 subunits. Earlier in this article we discussed that eIF3 can interact with YTHDF1 and increases translation efficiency. In 2015, Kate D. Meyer et al. first reported indicated that eIF3 can also directly binds to m^6^A in the 5′ UTR region of cellular mRNAs and recruit the 43 S ribosomal preinitiation complex to form 48 S initiation complexes and promote translation [53]. In addition, members of the heterogeneous nuclear ribonucleoprotein (hnRNP) family including hnRNPC, hnRNPG and hnRNPA2B1 were also found to act as m^6^A readers.

In 2015, N Liu et al. identified 39,060 m^6^A-switches in hnRNPC-binding sites. [54]. And in 2017, the same group further reported that hnRNPG can bind to m^6^A-modified RNAs via its C-terminal low-complexity region, which further functions in the regulation of gene expression and alternative splicing [55]. Functionally, hnRNPC and hnRNPG were found to be recruited to m^6^A modified mRNAs and lncRNAs to affect the absence and alternative splicing of target transcripts in a mechanism called “m^6^A-switch”, which means m^6^A affects RNA-protein interaction for biological regulation via controlling the RNA-structure-dependent accessibility of RNA binding motifs [54, 55]. Additionally, hnRNPG was proved to interact with m^6^A-modified nascent pre-mRNA and the phosphorylated C-terminal domain of RNA polymerase II via its RRM and Arg-Gly-Gly (RGG) motifs in the low-complexity region, which can regulate the alternative splicing of targeted RNAs [56].

In 2015, Claudio R. Alarcón et al. reported that hnRNPA2B1 can significantly bind to m^6^A marks primary-miRNA transcripts and promote primary-miRNA processing via interacting with the microRNA Microprocessor complex protein DGCR8 microprocessor complex subunit (DGCR8) [57]. There are also researches suggest that hnRNPA2B1 may also work through the “m^6^A switch” mechanism [58]. Moreover, proteins in insulin-like growth factor 2 mRNA-binding proteins (IGF2BPs) family, including IGF2BP1/2/3, are another important group of m^6^A readers that act in post-transcriptional gene regulation and tumorigenesis. In 2018, HL Huang et al. reported that IGF2BP1/2/3 can act as a novel m^6^A reader family that target mRNA transcripts via recognizing the consensus GG(m^6^A)C sequence [59]. The recognition of RNA m^6^A methylation by IGF2BPs can stabilize the structure of targeted mRNA and enhance translation, which acts a significant role in post-transcriptional gene regulation [59].

### m^6^A erasers

M^6^A methylation has been proved to be a reversible process, and this process mainly depends on the interaction between m^6^A writers and erasers. M^6^A erasers are a group of demethylases that can specifically remove m^6^A in targeted RNAs. FTO alpha-ketoglutarate dependent dioxygenase (FTO) was discovered as the first m^6^A eraser which has efficient oxidative demethylation activity targeting the abundant m6A residues in RNA in vitro in 2011 [60]. It can mediate demethylation in an Fe (II) and α-ketoglutaric acid-dependent manner. During m^6^A regulation, FTO can oxidize m^6^A into two additional modifications, N6-hydroxymethyladenosine (hm^6^A) and N6-formyladenosine (f^6^A), which are hard to be recognized by m^6^A readers. Finally, these two intermediates will be converted to adenosine (A), finishing the demethylation process [61]. In addition, alkB homolog 5 (ALKBH5) is the secondly identified m^6^A eraser. In 2013, GQ Zheng et al. presented ALKBH5 as a mammalian RNA demethylase that catalyzes the removal of the m^6^A modification on mRNA both in vitro and in vivo [62]. For the mechanism, ALKBH5 may also act in the iron center-catalyzed oxidative demethylation way, but the detailed mechanism still needs a further investigation in the future [62].

## Biogenesis and biological functions of lncRNAs

As mentioned before, lncRNAs can be generally defined as a class of non-coding RNAs more than 200 nt in length with or without protein coding ability [6, 7]. Before we discuss the interaction between m^6^A methylation and lncRNAs, some basic information about the biogenesis and functions of lncRNAs can be necessary.

### Biogenesis

Basically, lncRNAs can be divided into five categories, including intronic lncRNAs, intergenic lncRNAs sense lncRNAs and antisense lncRNAs. Intronic lncRNAs and intergenic lncRNAs are respectively transcribed from intron sequences in genes and protein-coding intergenic sequences [63, 64]. Sense lncRNAs are transcribed from minor promoter in protein-coding sequences, while the transcription of antisense lncRNAs is initiated by the single-stranded DNA (ssDNA) of R-loop [63–65]. As widely known, transcription of the eukaryotic genome is carried out by three main RNA polymerases, including RNA polymerase I (Pol I), Pol II and Pol III, most lncRNAs are transcribed by Pol II [63]. The transcription of lncRNA is similar with mRNA, which needs the cooperation of Pol II and general transcription factors (TF) TFIIA, TFIIB, TFIID, TFIIE, TFIIF and TFIIH to form the pre-initiation complex (PIC) [66]. For initiation, the TATA-binding protein (TBP) in TFIID first recognizes and binds to the TATA box in promoter sequences with the help of several TBP-associated factors (TAFs). Meanwhile, TFIIF and Pol II will form a stable Pol II–TFIIF complex. Then, TFIIB will bind to TBP and form a TFIIB–TBP–DNA promoter complex. TFIIA can help to stabilize the structure of this complex during this process. Afterwards, the Pol II–TFIIF complex and TFIIB–TBP–DNA promoter complex will link together, resulting in the formation of the core PIC. Finally, TFIIF and TFIIH will be recruited to this complex to form the closed PIC. TFIIH has helicase activity, which enables the cleavage of DNA double helix near the transcription start sites, leading to the formation of the opened PIC and a transcription bubble, and LncRNA or mRNA will be transcribed later [67, 68].

### Biological functions

LncRNA can act by regulating cellular processes at various levels. Basically, the biological functions of lncRNAs can be divided into five parts, including epigenetic modification, transcriptional regulation, post-transcriptional regulation, translational regulation and post-translational regulation [69]. (Table 1)

**Table 1:** Brief biological functions of lncRNA. What should be noticed is that some of these lncRNAs have multiple functions and mechanisms, this table just listed the functions and mechanisms mentioned in our review

LncRNAs	Functions	Mechanisms	References
XIST	Epigenetic modification	Recruit chromatin modification factors	[70, 71]
HOTAIR	Epigenetic modification	Recruit chromatin modification factors	[72]
LncPRESS1	Epigenetic modification	Interdict the recruitment of some chromatin modifiers	[73]
XIST	Transcriptional regulation	Modulate RNA polymerases	[74]
SLERT	Transcriptional regulation	Modulate RNA polymerases	[75]
Khps1	Transcriptional regulation	Form R-loops	[77]
1/2-sbsRNAs	Post-transcriptional regulation	Recruit degradation proteins	[78]
LncIRS1	Post-transcriptional regulation	Compete for microRNA binding	[79]
SINEUP- GFP, SINEUP-ΔAlu	Translational regulation	Modulate translational initiation complexes	[82]
CCAT2	Translational regulation	Interact with ribosomes and initiation factors	[83]
DILA1	Post-translational regulation	Inhibit the phosphorylation of proteins and block its subsequent degradation	[84]

#### Epigenetic modification

LncRNAs can recruit chromatin modification factors and direct them to their target gene sites, leading to epigenetic modifications of DNA or histone. For this part, one of the best-studied lncRNAs is XIST, which acts a significant role in transcriptional silencing of one X-chromosome during development in female mammals [70]. XIST accumulates on the X-chromosome in cis and can directly recruit polycomb repressive complex 2 (PRC2), leading to accumulation of histone H3 lysine 27 trimethylation (H3K27me3) and X-chromosome inactivation (XCI) [71]. Another example, homeobox (HOX) transcript antisense RNA (HOTAIR), can coordinate histone modifications by binding to multiple histone modification enzymes. The 5′ domain of HOTAIR binds PRC2 while the 3′ domain of HOTAIR binds lysine specific demethylase 1 (LSD1), resulting in coordinated targeting of PRC2 and LSD1 to chromatin for combined histone H3 lysine 27 methylation and lysine 4 demethylation [72].

In addition to recruit chromatin modification factors, lncRNAs can also interdict the recruitment of some chromatin modifiers. For example, lncRNA p53 regulated and ESC associated 1 (lncPRESS1) can interact with sirtuin 6 (SIRT6) and interdict its chromatin localization. As SIRT acts as a deacetylase, which can reduce the acetylation levels of Hst3 histone H3 K56 (H3K56) and H3K9, the interaction between lncPRESS1 and SIRT6 maintains high acetylation levels of histone H3K56 and H3K9 at promoters of pluripotency genes, activating transcription [73]. Interestingly, lncPRESS1 is also a p53-regulated transcript, which indicates a new function mechanism of p53 pathway [73].

#### Transcriptional regulation

LncRNAs can regulate gene transcription by modulating RNA polymerases. For instance, XIST can directly interact with silencing mediator for retinoid and thyroid hormone receptor (SMART)/histone deacetylase 1 (HDAC1)-associated repressor protein (SHARP). This interaction can recruit SMART, activate HDAC1 and deacetylate histones to exclude Pol II across the X-chromosome, leading to transcriptional silencing [74]. In addition to Pol II, lncRNAs can also modulate Pol I. YH Xing et al. reported a box H/ACA small nucleolar RNA (snoRNA)-ended lncRNA that enhances pre-rRNA transcription (SLERT). SLERT can interact with DEAD-box helicase 21 (DDX21) and evict DDX21 suppression on Pol I machinery, resulting in enhanced pre-rRNA transcription [75].

Additionally, lncRNAs can modulate gene transcription via forming R-loops. R-loops are triple-stranded nucleic acid structures with RNA hybridized to duplex DNA [76]. LncRNAs can participate in the formation of R-loops and then recruit transcription cofactors to transcription initiation regions. For example, lncRNA Khps1 is transcribed in antisense orientation to the proto-oncogene sphingosine kinase 1 (SPHK1). Then Khps1 can interact with a homopurine stretch upstream of the transcription start site of SPHK1 to form a DNA-RNA triplex or R-loop. This R-loop formed with Khps1 and SPHK1 can anchor associated effector proteins to the transcription initiation regions, leading to augmented transcription [77].

#### Post-transcriptional regulation

LncRNA can influence the structure and stability of message RNAs (mRNAs) in several ways. First, lncRNAs can recruit proteins that are associated with mRNA degradation to regulate mRNA stability. For instance, CG Gong et al. reported a group of lncRNAs named half-STAU1-binding site RNAs (1/2-sbsRNAs), which can interact with target mRNAs to form double-stranded RNAs (dsRNAs). This structure will be identified by staufen 1 (STAU1), leading to a STAU1-mediated mRNA degradation [78]. Second, some lncRNAs are also known as competitive endogenous RNAs (ceRNAs), which can regulate mRNA expression via competing for microRNA (miRNA) binding. For example, lncRNA insulin receptor substrate 1 (lncIRS1) acts a ceRNA of miR-15a, miR-15b-5p and miR-15c-5p to upregulate insulin receptor substrate 1 (IRS1) expression [79]. Moreover, the lncRNA-miRNA-mRNA axis has been found in the progression of several cancers, like osteosarcoma and gastric cancer [80, 81], indicating a significant role in cancer detection and treatment.

#### Translational regulation

LncRNA can modulate the formation of translational initiation complexes to mediate translational regulation. For example, SINEUPs are lncRNAs with a SINE element, which can up-regulate the translation of target mRNA. Naoko Toki et al. reported that SINEUP- GFP and SINEUP-ΔAlu co-localized with mRNA EGFP in the cytoplasm and recruit polypyrimidine tract binding protein 1 (PTBP1) and hnRNPK, which contributed to assembly of translational initiation complexes, resulting in enhanced EGFP mRNA translation [82]. Moreover, lncRNAs are also known to interact with ribosomes and initiation factors to regulate translation. For instance, lncRNA colon cancer associated transcript 2 (CCAT2) could straightly stabilize BOP1 ribosomal biogenesis factor (BOP1), which increased the expression of genes involved in ribosome biogenesis, and further led to increased active form of aurora kinase B (AURKB) [83].

#### Post-translational regulation

For the last part, lncRNA can influence the stability of proteins to form post-translational regulation in cells. For example, Cyclin D1 is one of the most important oncoproteins that acts an important role in breast cancer cell proliferation and tamoxifen resistance. QF Shi et al. reported a lncRNA DILA1 which can inhibit the phosphorylation of Cyclin D1 at Thr286 by directly interacting with Thr286 and blocking its subsequent degradation. This interaction resulted in overexpression of Cyclin D1, leading to lower tamoxifen sensitivity in breast cancer cells [84].

## Mechanisms of m^6^A-lncRNAs mediated cancer regulation

**Fig. 3:**
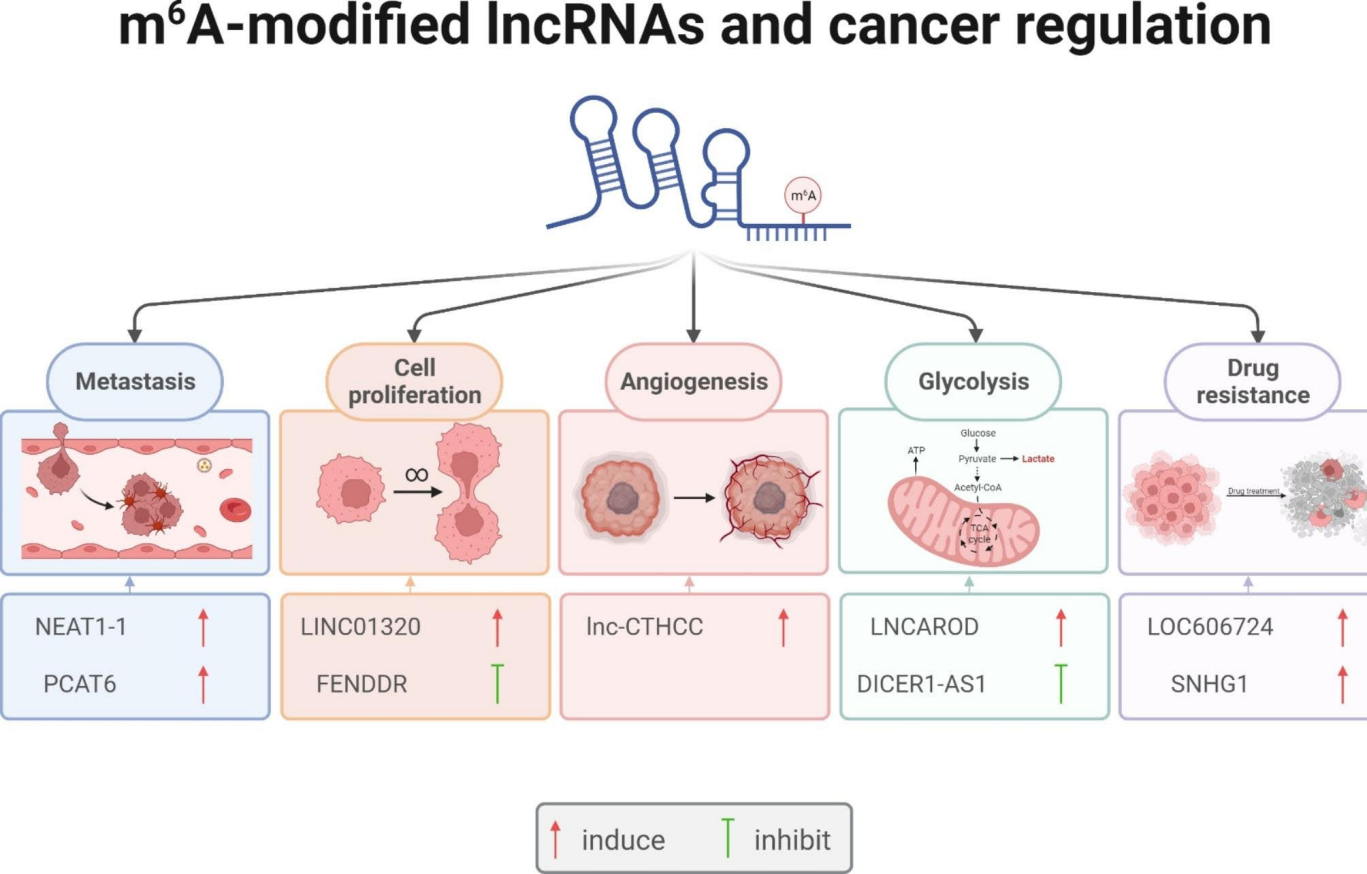
Mechanisms of m^6^A-lncRNAs mediated cancer regulation. M^6^A-midified lncRNAs can regulate cancer progression in several mechanisms, including regulating metastasis, cell proliferation, angiogenesis, glycolysis and drug resistance

With the development of MeRIP-m^6^A-seq technology, lots of m^6^A-modified lncRNAs were identified and their unique biological functions were gradually clarified, which has aroused wide attention in clinical research and medical development, especially in the oncology field. Till now, growing evidence suggests that m^6^A-modified lncRNAs affect cancer progression via several mechanisms, including regulating metastasis, cell proliferation, angiogenesis, glycolysis and drug resistance (Fig. 3).

### Metastasis

Metastasis is a common characteristic of malignant tumors, which often occurs very early but takes years to develop overt clinical manifestations [85].

Growing facts revealed that m^6^A-modified lncRNAs acted a significant role in regulating cancer metastasis. Prostate cancer is a common malignancy among males, accounting for the second leading cause of cancer-related mortality [86]. For these patients, metastasis is the main cause of death, and bone is the most common distant metastatic organ [87]. M^6^A-modified lncRNAs have been proved to participate in the prostate cancer bone metastasis. For instance, SM Wen et al. reported an upregulated lncRNA nuclear-enriched abundant transcript 1 (NEAT1-1) in prostate cancer tissues, of which m^6^A modification level was significantly elevated. M^6^A RIP-seq and data analysis showed that there were four credible m^6^A sites on NEAT1-1 (labeled as #1 to #4 from 5′ to 3′ of NEAT1-1, #1: UUGGACUUUUC, #2: AGAACAUU, #3: AAUAACUUG, #4: AAUGACUUGG), high m^6^A level of NEAT1-1 was a powerful predictor of bone metastasis and eventual death. Further mechanism research revealed that NEAT1-1 could interact with cyclin L1 (CCNL1) through its #4 m^6^A and acted as a bridge between CCNL1 and cyclin dependent kinase 19 (CDK19) to form a stable complex, which may be specific in in bone metastatic prostate cancer. Afterwards, via the m^6^A site #3, NEAT1-1 could recruit this CCNL1/CDK19/NEAT1-1 complex to RUNX family transcription factor 2 (RUNX2) promoter, which further activated RNA Polymerase II (RNPII) serine2 phosphorylation and promoted RUNX2 expression. As RUNX2 is a widely known vital marker and driver in bone metastatic prostate cancer, this CCNL1/CDK19/NEAT1-1 complex can significantly induce the bone metastasis of prostate cancer [88].

Additionally, lncRNA prostate cancer associated transcript 6 (PCAT6) was also found to promote bone metastasis in prostate cancer. PCAT6 is upregulated in cancer tissues with bone metastasis, increased PCAT6 expression predicates poor prognosis in prostate cancer patients. In terms of mechanism, m^6^A methylation was proved to be the cause of PCAT6 upregulation. METTL3 mediates m^6^A modification on PCAT6 and IGF2BP2 then identifies these modifications and promotes PCAT6 expression. In addition, overexpressed PCAT6 can then form an RNA-protein three-dimensional complex together with IGF2BP2 and insulin like growth factor 1 receptor (IGF1R) mRNA, which enhances IGF1R mRNA stability and contributed to IGF1R upregulation. Then IGF1R could participate in the activation of PI3K/AKT and NF-κB signaling, leading to induced bone metastasis [89].

Moreover, Xinyu Wang et al. reported another lncRNA metastasis-associated lung adenocarcinoma transcript 1 (MALAT1) that was proved to enhance the metastasis ability of esophageal cancer (ESCC). MALAT1 was found to specifically localize in nuclear speckles (NSs). M^6^A writer RBM15 could embed into NSs to form stable interactions with MALAT1 and further mediated continuously depositing m^6^A methylation onto MALAT1. Then m^6^A reader YTHDC1 recognized m^6^A-modified MALAT1 and this recognition enriched majority of the reduced proteins (37 out of 39) in NSs, which regulated the homeostasis of NSs and further engendered transcriptome reprogram, restoring migration ability [90].

### Cell proliferation

As a basic feature of cancer cells, uncontrollable cell proliferation is one of the leading causes of malignant biological behaviors of cancers. LncRNAs have been proved to be critical for cancer cell survival and proliferation, like lncRNA brain cytoplasmic RNA 1 (BCYRN1 or BC200) was found to be dramatically upregulated in cultured tumor cells than normal cells, and knockdown of BC200 significantly inhibit the viability of cancer cells via arresting cell cycle and inducing apoptosis [91].

With the synergistic effect of m^6^A methylation, m^6^A-modified lncRNAs serve more importantly in cell proliferation. For instance, m^6^A-mediated upregulation of lncRNA LINC01320 was found to promote the proliferation, migration, and invasion of gastric cancer. M^6^A methylation mediated by m^6^A writer METTL14 significantly induces the enrichment of LINC01320. Enriched LINC01320 then directly targets miR-495-5P, resulting in downregulation of miR-495-5p. Further, the downregulation of miR-395-5P removes the silencing of gen RAB19, member RAS oncogene family, leading RAB19 overexpression in GC tissues, which promotes the cell proliferation, migration, and invasion of GC cells [92].

In addition, m^6^A regulated lncRNA FOXF1 adjacent non-coding developmental regulatory RNA (FENDDR) was also found to regulate the cell proliferation of cancer cells, which is downregulated in endometrioid endometrial carcinoma (EEC) tissues. FENDDR is a well-known tumor-suppressor gene, overexpressed FENDDR can inhibit the expression of SRY-related HMG box transcription factor 4 (SOX4) to suppress cell proliferation and facilitate cell apoptosis of EEC cells. As the m^6^A methylation levels of FENDRR is increased in cancerous tissues of EEC patients, researches proved that the expression level of LncRNA FENDRR was negatively regulated by m^6^A modification. M^6^A modifications on FENDRR recruit m^6^A reader YTHDF2, which promotes FRNDRR degradation, and YTHDF2 knockdown significantly suppresses the cell proliferation of EEC cells by reducing m^6^A-mediated FENDRR degradation [93].

### Angiogenesis

An abnormal vascular network is essential for tumorigenesis. During cancer progression, cancer cells keep secreting high levels of angiogenic factors to induce a special vascular system consists of disorganized, immature and permeable blood vessels, which helps to form a hypoxic microenvironment around cancer tissues [94]. Cancer-testis (CT) genes are a class of gene that participates in the initiation and progression of cancer. Normally, CT genes only express in the testes, but during tumorigenesis, CT genes are reactivated and turn into a high expression pattern [95].

Recently, a conserved CT-lncRNA named lnc-CTHCC was first discovered, which was highly expressed in the hepatocellular carcinoma (HCC) and was proved to promote angiogenesis in HCC progression [96]. A lnc-CTHCC-knockout mouse model showed that the loss of lnc-CTHCC inhibits the development of HCC, and both in vitro and in vivo assays confirmed that overexpressed lnc-CTHCC induces HCC angiogenesis. In terms of mechanism, m^6^A methylation leads to lnc-CTHCC upregulation. METTL3 mediates m^6^A methylation on lnc-CTHCC and IGF2BP1/IGF2BP3 then recognizes the m^6^A modification, which maintains lnc-CTHCC stability and increases its expression in HCC. Upregulated lnc-CTHCC then contacts and tethers hnRNPK to the Yes1 associated transcriptional regulator (YAP1) promoter for its activation and activated YAP1 is essential in the malignant phenotype of HCC cells. In conclusion, this METTL3–IGF2BP1/IGF2BP3–lnc-CTHCC–hnRNPK–YAP axis significantly promotes hepatocellular carcinogenesis and progression via inducing angiogenesis [96].

### Glycolysis

It has been widely known that tumor cells often use a special energy metabolism way that is significantly different from that of normal cells. Most cancer cells produce energy not through the usual citric acid and oxidative phosphorylation, but predominantly through a less efficient process of aerobic glycolysis. This observation was first found by Otto Heinrich Warburg and so was called as Warburg effect [97].

LncRNA activating regulator of DKK1 (LNCAROD), also known as LINC01468 or lnc-MBL2-4, was significantly upregulated and was found to enhance glycolysis in HCC patients. In HCC progression, METTL3/IGF2BP1-mediated m^6^A modification is pivotal in the upregulation of LNCAROD. METTL3 mediates m^6^A methylation on LNCAROD and IGF2BP1 then recognize the modification on LICAROD to maintain its stability [98]. Overexpressed LNCAROD then upregulates the level of pyruvate kinase M2 (PKM2) by interacting with serine-and arginine-rich SRSF3, which is a splicer of PKM that induces PKM switching from PKM1 to PKM2. Meanwhile, LNCAROD can also maintain PKM2 levels in HCC by acting as a ceRNA against miR-145-5p, which sponging activity inhibits PKM2 activity. Upregulated PKM2 then leads to enhanced glycolysis, resulting in HCC malignancy [98].

Moreover, another lncRNA called antisense RNA1 of DICER1 (DICER1-AS1), which is downregulated in pancreatic cancer (PC) tissues and overexpression of DICER1-AS1 inhibits glycolysis, proliferation and metastasis of PC cells both in vitro and in vivo. Mechanistic assays showed that m^6^A methylation leads to the downregulation of DICER1-AS1. M^6^A reader YTHDF3 decreases DICER1-AS1 stability in a m^6^A dependent manner. Additionally, in PC cells, DICER1-AS1 can recruit transcription factor YIN‑YANG‑1 (YY1) to the DICER1 promoter, which results in promoted transcription of DICER1. DICER1 then promotes the maturation of miR-5586-5p, which regulates glycolysis by inducing mRNA degradation of glycolytic genes, including lactate dehydrogenase A (LDHA), hexokinase 2 (HK2), phosphoglycerate kinase 1 (PGK1) and solute carrier family 2 member 1 (SLC2A1). Taken together, this YTHDF3/DICER1-AS1/DICER1/miR-5586-5p axis acts a pivotal role in glycolysis and tumorigenesis of PC [99].

### Drug resistance

Due to the rapid multiplication rate, it is easy for cancer cells to form drug resistance to therapy, which severely weakens the efficacy of treatment. Clarifying the mechanism of how cancer cells form drug resistance can be urgent and valuable for cancer treatment [100].

Recently, m^6^A-modified lncRNAs have been found to be associated with the construction of drug resistance. For example, lncRNAs in adipocytes-secreted exosomes of multiple myeloma (MM) patients were found to protect MM cells from the apoptosis induced by chemotherapy and raised LncRNA levels in MM cells were positively correlated to poor prognosis in MM patients. Further studies found that two lncRNAs, coronin 1 A pseudogene LOC606724 and small nucleolar RNA host gene 1 (SNHG1), are significantly upregulated in MM cells after being secreted into adipocyte exosomes. Mechanistically, MM cells promote enhancer of zeste 2 polycomb repressive complex 2 subunit (EZH2)-mediated METTL7A protein methylation and enhance METTL7A activity in adipocytes. METTL7A then mediates m^6^A methylation on LOC606724, SNHG1 and other lncRNAs, which promotes lncRNAs package into adipocyte exosomes and indices MM drug resistance [101].

## m^6^A modified lncRNAs in cancers

Thus far, lots of studies have shown that m^6^A methylation can significantly regulate the progression of various cancers. This regulation often starts with the m^6^A modifications catalyzed by m^6^A writers in the mRNAs of oncogenes or tumor suppressors, and then these marks can be recognized by m^6^A readers and result in upregulation or downregulation of these genes. Meanwhile, m^6^A can be removed by m^6^A erasers from the mRNAs of oncogenes or tumor suppressors, thus averting the acting of m^6^A readers, leading to upregulation or downregulation of these genes [102]. Except mRNAs, lots of evidence have shown that lncRNAs also cooperate with m^6^A methylation and regulate cancer progression. Next, we briefly reviewed the m^6^A modified lncRNAs in human cancers by system (Table 2).

**Table 2:** The multifaceted roles of M^6^A-modified lncRNAs in human cancers

Cancers	M^6^A-modified lncRNAs	Expression level	Functions	References
Colorectal cancer	XIST	Upregulated	Promote cell proliferation and metastasis	[107]
	GAS5	Downregulated	Inhibit cancer progression	[108]
	RP11	Upregulated	Promote metastasis	[110]
Gastric cancer	LINC01320	Upregulated	Promote cell proliferation	[92]
	THAP7-AS1	Upregulated	Promote metastasis and cell proliferation	[113]
Esophageal squamous cell carcinoma	LINC00022	Upregulated	Promote cell proliferation	[116]
	MALAT1	/	Promote metastasis	[90]
Hepatocellular carcinoma	lnc-CTHCC	Upregulated	Promote angiogenesis	[96]
	LNCAROD	Upregulated	Promote glycolysis	[98]
	LINC00958	Upregulated	Facilitate lipogenesis	[118]
Pancreatic cancer	DICER1-AS1	Downregulated	Inhibit glycolysis	[99]
	WTAPP1	Upregulated	Promote cell proliferation	[122]
	KCNK15-AS1	Downregulated	Inhibit cell proliferation	[123]
Nasopharyngeal carcinoma	ZFAS1	Upregulated	Regulate autophagy level	[127]
	FAM225A	Upregulated	Promote cell proliferation	[128]
Lung cancer	MALAT1	Upregulated	Promote drug resistance and metastasis	[135]
	ABHD11-AS1	Upregulated	Promote glycolysis	[136]
	AC138035.1 et al.	/	/	[138]
	SVIL-AS1	Downregulated	Inhibit cell proliferation	[142]
Prostate cancer	NEAT1-1	Upregulated	Promote metastasis	[88]
	PCAT6	Upregulated	Promote metastasis	[89]
Cervical cancer	KCNMB2-AS1	Upregulated	Facilitate cancer progression	[144]
	FOXD2-AS1	Upregulated	Facilitate cancer progression	[145]
Endometrial carcinoma	FENDDR	Downregulated	Inhibit cell proliferation	[93]
	AL645568.1 et al.	/	/	[148]
Ovarian cancer	AC010894.3 et al.	/	/	[150]
	AC008669.1 et al.	/	/	[151]
	RHPN1-AS1	Upregulated	Promote cell proliferation and metastasis	[152]
Bladder cancer	AC020911.1 et al.	/	/	[155]
	ZNRD1-AS1 et al.	/	/	[156]
Renal cell carcinoma	NEAT1	Downregulated	Inhibit cell proliferation	[158]
	Lnc-LSG1	Downregulated	Inhibit metastasis	[159]
Thyroid cancer	HAGLR	Upregulated	Promote cell proliferation	[162]
	MALAT1	Upregulated	Promote cell proliferation	[164, 165]
	PSMG3-AS1 et al.	/	/	[167]
	AC139795.2 et al.	/	/	[168]
Breast cancer	MIR210HG	Upregulated	Promote cell proliferation	[172]
	LINC00958	Upregulated	Promote cell proliferation	[173]
Multiple myeloma	LOC606724	Upregulated	Promote drug resistance	[101]
	SNHG1	Upregulated	Promote drug resistance	[101]
Lymphoma	TRERNA1	Upregulated	Promote cell proliferation	[177]
Leukemia	NEAT1	Downregulated	Promote apoptosis	[180]
	AC025430.1 et al.	/	/	[182]
Osteosarcoma	ZBTB32 et al.	/	/	[186]
	AP003119.2 et al.	/	/	[187]
	FOXD2-AS1	Upregulated	Promote cell proliferation and metastasis	[188, 189]
	PVT1	Upregulated	Promote cell proliferation	[190–193]
Skin cutaneous melanoma	RP11-775D22.3 et al.	/	/	[195]
Glioma	C6orf3 et al.	/	/	[198]
Glioblastoma	CASC9	Upregulated	Promote glycolysis	[200]
Head and neck squamous cell carcinoma	GRHL3-AS1 et al.	/	/	[203]
	LNCAROD	Upregulated	Promote cell proliferation	[204]
Oral squamous cell carcinoma	AC079684.2 et al.	/	/	[206]
	MALAT1	Upregulated	Promote cell proliferation	[207]
Laryngeal squamous cell carcinoma	KCNQ1OT1	Upregulated	Promote cell proliferation	[209]

### Gastrointestinal cancers

Gastrointestinal cancer refers to a malignant condition of the gastrointestinal tract and accessory organs of digestion, including the esophagus, stomach, biliary system, pancreas, small intestine, large intestine, rectum and anus [103]. Thus far, a number of studies have indicated the experience of m^6^A modified lncRNAs in colorectal cancer (CRC), gastric cancer, esophageal squamous cell carcinoma (ESCC), HCC, and PC.

#### Colorectal cancer

CRC is the most common gastrointestinal cancer, which ranked third in the global top 10 cancers in 2015 and caused 832 000 deaths worldwide [104]. Till now, colonoscopy is still the method of choice for CRC diagnosing. However, early CRCs often appear as subtle mucosal lesions, which may be blurry in colonoscopy [105]. To ensure detection, more laboratory detections are essential, and m^6^A-modified lncRNAs may serve as effective biomarkers. For example, lncRNA XIST has been shown to act a significant role in the proliferation and metastasis of CRC [106]. Meanwhile, m^6^A mapping studies have indicated that XIST was highly methylated with at least 78 m^6^A residues [35]. Recent evidence proved that m^6^A writer METTL14 and m^6^A reader YTHDF2 directly participated in the m^6^A methylation and regulation of XIST in CRC. METTL14 mediated m^6^A methylation in XIST and TYHDF2 further recognized m^6^A-methylated XIST to mediate XIST degradation. In a word, XIST expression negatively correlates with METTL14 and YTHDF2 in CRC [107].

LncRNA growth arrest-specific 5 (GAS5) was also proved to cooperate with m^6^A methylation to regulate CRC progression by mediating post-translational regulation of YAP [108]. GAS5 functions in the Hippo/Yes-associated protein (YAP) pathway, which is required to drive tumor initiation and progression [109]. In CRC, GAS5 was localized in both the cytoplasm and the nucleus while YAP was localized in in the nucleus. GAS5 could directly interact with the WW domain of YAP to mediate translocation of YAP from the nucleus to the cytoplasm. Afterwards, GAS5 stimulated YAP phosphorylation to facilitate its ubiquitination and degradation to inhibit colorectal cancer progression. In this process, m^6^A reader YTHDF3 can selectively bind to m^6^A-modifed GAS5 and mediate GAS5 degradation in a methylation dependent manner, which leads to dysregulation of GAS5 and YAP accumulation in CRC progression [108].

Another lncRNA that is highly expressed in CRC tissues is RP11-138 J23.1 (RP11), whose expression increased with CRC stage in patients. In CRC, m^6^A-modified RP11 functioned through forming a complex with m^6^A reader hnRNPA2B1 and mRNA. This RP11/hnRNPA2B1/mRNA complex significantly induced the mRNA degradation of two E3 ligases, siah E3 ubiquitin protein ligase 1 (Siah1) and F-box protein 45 (Fbxo45), which further inhibited the degradation of zinc finger E-box binding homeobox 1 (Zeb1), leading to enhanced CRC metastasis. Moreover, the m^6^A methylation also involved in the upregulation of RP11 via promoting its nuclear accumulation. This m^6^A/RP11/Zeb1 axis significantly triggered the progression of CRC in vivo [110].

#### Gastric cancer

**Fig. 4:**
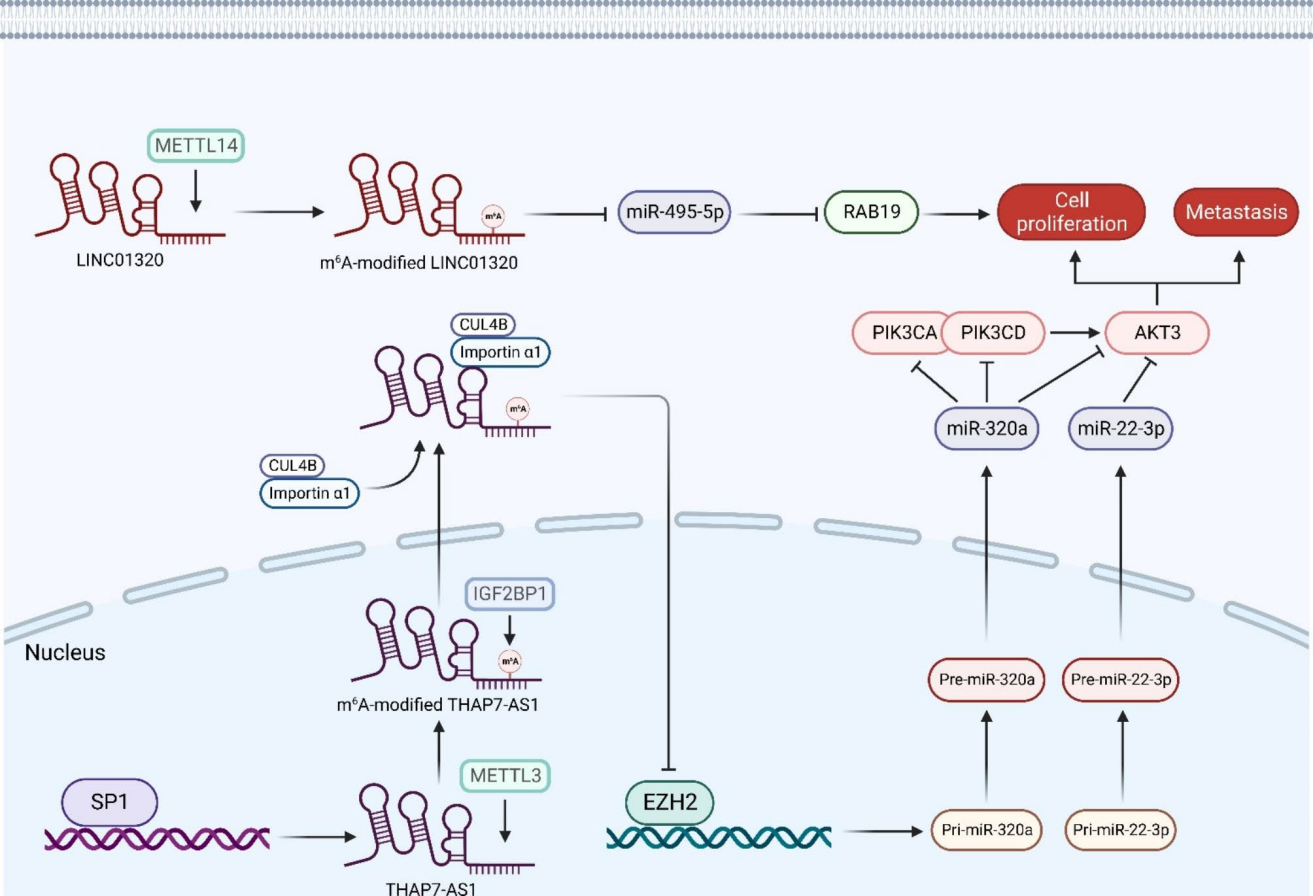
M^6^A-modified lncRNAs in gastric cancer. LINC01320 promotes GC cell proliferation via METTL14/LINC01320/miR-495-5p/RAB19 axis; THAP7-AS1 is modified by METTL3, stabilized by IGF2BP1 and mediates CUL4B nucleus transportation, which further inhibits the expression of miR-320a and miR-22-3P, leading to promoted GC cell proliferation and metastasis

Gastric cancer is the sixth most commonly diagnosed cancer and the third leading cause of cancer death worldwide, with 1,033,701 new cases and 782,685 deaths in 2018 [111]. H pylori infection is the most leading risk factor for non-cardia GC, and older age, smoking, alcohol ingesting, family disease history and previous gastric surgery are also cofactors [112]. In recent years, liquid biopsies have raised lots of attentions in GC pathology, and results from circulating tumor DNA (ctDNA) have been most promising in GC patients [112]. As another potential biomarkers of liquid biopsies, m^6^A-modified lncRNAs may also provide a novel insight in GC diagnosing. For instance, lncRNA THAP7 antisense RNA 1 (THAP7-AS1) was proved to be considerably upregulated in gastric cancer tissues compared with healthy stomach tissues. In gastric cancer cells, THAP7-AS1 specifically interacted with importin α1 and the nuclear localization signal region of cullin 4B (CUL4B) and mediated the entry of THAP7-AS1/CUL4B complex into the nucleus. The nuclear localization of THAP7/CUL4B complex then transcriptionally repressed EZH2-mediated expression of miR-22-3p/miR-320a and further initiated phosphatidylinositol 3-kinase/AKT serine/threonine kinase (PI3K/AKT) signaling pathway via promoting the expression of phosphatidylinositol-4,5-bisphosphate 3-kinase catalytic subunit alpha (PIK3CA), phosphatidylinositol-4,5-bisphosphate 3-kinase catalytic subunit delta (PIK3CD) and AKT3, leading to enhanced cell proliferation and metastasis. During the biosynthesis of THAP7-AS1, Sp1 transcription factor (SP1) bound directly to the THAP7-AS1 promoter region to activate its transcription, and m^6^A methylation mediated by m^6^A writer METTL3 induced THAP7-AS1 expression via a IGF2BP1-dependent way, which donates in the metastasis of gastric cancer [113]. (Fig. 4)

#### Esophageal squamous cell carcinoma

ESCC is a subtype of esophageal cancer (ESCA), which is one of the most fatal malignancies around the world. In China, ESCC accounts for more than 90% of all ESCA patients [114]. Though the global incidence of ESCA gradually declined along with the medical advances, the annual global mortality rate of ESCA still ranks sixth in cancer induced death, with 544,076 new deaths in 2020 [115]. M^6^A eraser FTO was found to overexpressed in ESCC tissue and associated with tumor growth. MeRIP-m^6^A-seq analysis combined with bioinformatics analysis indicated that lncRNA LINC00022 is the down-stream target of FTO. In ESCC cells, FTO mediated the m^6^A demethylation of LINC00022 and further promoted LINC00022 expression in an YTHDF2-dependent manner. Upregulated LINC00022 then induced the ubiquitination of p21 protein and negatively regulated the protein level of p21 through the ubiquitin-proteasome pathway, leading to promoted cell proliferation and tumor growth of ESCC [116].

#### Hepatocellular carcinoma

**Fig. 5:**
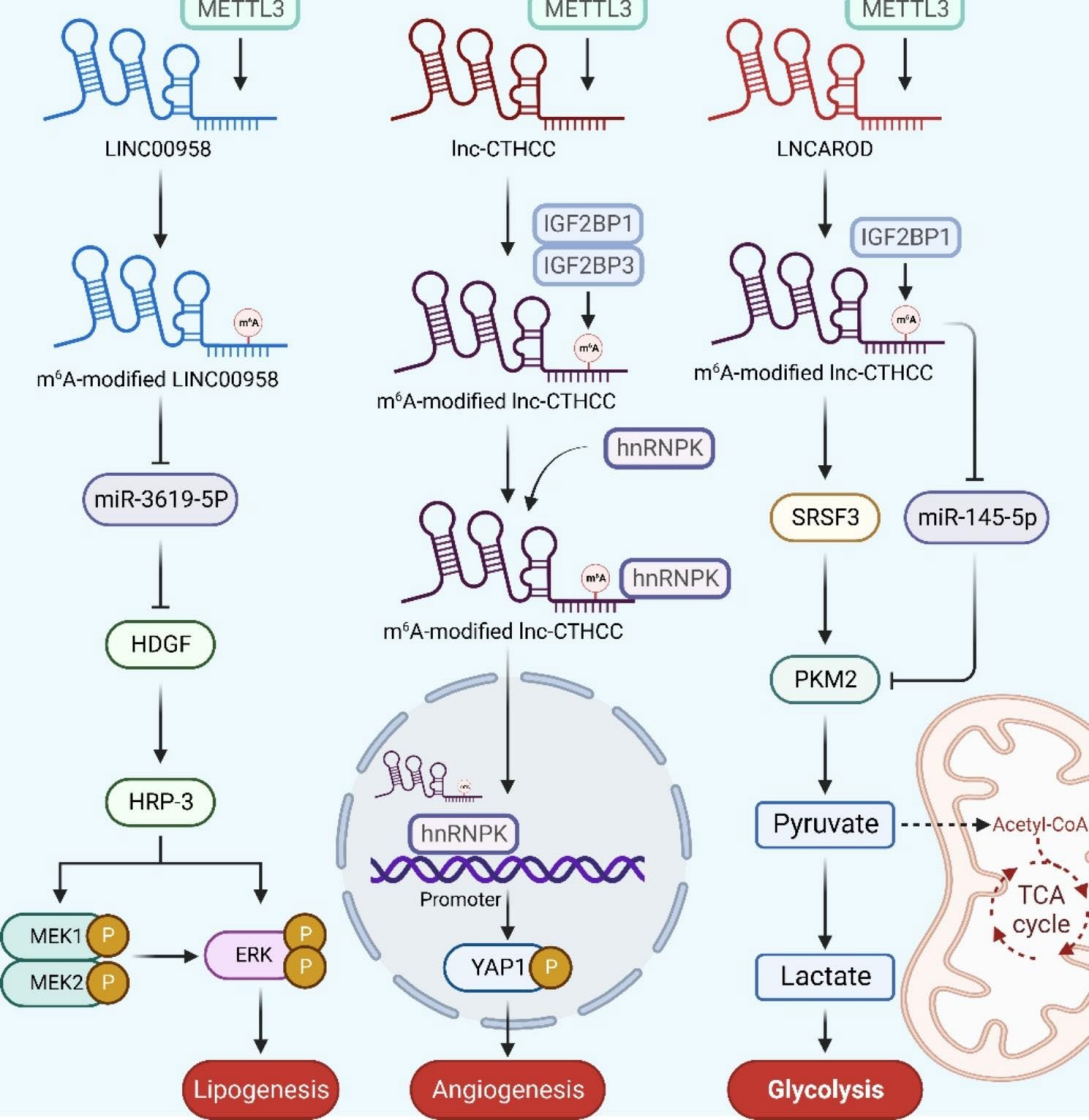
M^6^A-modified lncRNAs in HCC. LINC00958 promotes HCC lipogenesis via METTL3/LINC00958/miR-3619-5P/HDGF/HRP-3/MEK1/2/ERK axis; METTL3-mediated m^6^A-modified Lnc-CTHCC functions by locating hnRNPK to the promoter of YAP1 gene, leading to promoted HCC angiogenesis; LINAROD is modified by METTL3 and stabilized by IGF2BP1, which functions by both inducing SRSF3 and inhibiting miR-145-5P to induce PKM2, leading to promoted HCC glycolysis

With 905,677 new cases and 830,180 new deaths in 2020, HCC ranks the seventh most commonly diagnosed malignancy and the third leading cause of tumor death worldwide [115]. For HCC diagnosing, the preferred test is ultrasonography, but its diagnostic accuracy can be unsatisfactory due to unskilled operators. Serum tumor markers, most commonly α-fetoprotein, are an attractive alternative for HCC surveillance and early diagnosis, but false-positive suspicions are still high [117]. Therefore, identifying another biomarker is valuable, and m^6^A-modified lncRNAs may have a great potential. Recently, lncRNA LINC00958 has been found to be upregulated in HCC. LINC00958 was essential for malignant behaviors in HCC cells and high LINC00958 level significantly indicated poor survival rate. Mechanistic studies showed that LINC00958 targeted miR-3619-5p to utilize its tumor promoting effects in HCC, and hepatoma-derived growth factor (HDGF), a direct target of miR-3619-5p, was also vital for the function of LINC00958. In HCC cells, overexpressed LINC00958 sponged miR-3619-5P to upregulate HDGF, which further facilitates HCC lipogenesis and progression [118]. HDGF is a widely-known cancerogenic protein in HCC, HDGF-related protein-3 (HRP-3) has been proved to promote the phosphorylation of mitogen activated kinase-like protein (MAPK) and extracellular regulated MAP kinase (ERK), leading to activated MAPK/ERK signaling pathway and facilitated HCC progression [119]. Moreover, in this LINC00958/miR-3619-5p/HDGF axis, m^6^A methylation was also found to act an important role in LINC00958 upregulation. M^6^A methylation mediated by METTL3 stabilized RNA transcript of LINC00958, resulting in LINC00958 upregulation [118]. (Fig. 5) As m^6^A-modified LINC00958 plays an essential role in HCC progress, XL Zu et al. developed a novel PLGA-based nanoplatform encapsulating si-LINC00958 for HCC systemic administration. This novel system showed satisfactory antitumor efficacy in HCC PDX models, indicating its great value as a nanotherapeutic candidate in HCC [118].

#### Pancreatic cancer

Pancreatic cancer (PC) is a malignant digestive system cancer with a distinct microenvironment and high mortality rate. With 495,773 new cases and 466,003 new deaths in 2020, PC keeps being a leading cause of cancer death worldwide, which lobal burden has more than doubled over the past 25 years [115, 120]. Until then, Tri-phasic pancreatic-protocol CT is the best preliminary diagnostic test for PC. High-quality pancreatic-protocol CT scans can effectively detect locally advanced and metastatic disease [121]. Although imaging examination can strongly reveal PC, laboratory diagnosis is still recommended to rule out some benign disorders and PC molecular markers still need further evaluation. Lately, lncRNA Wilms tumor 1 associated protein pseudogene 1 (WTAPP1) was identified to be significantly elevated in pancreatic ductal adenocarcinoma (PDAC), one of the most common subtypes of PC, and was associated with poor prognosis. Overexpression of WTAPP1 significantly induced PDAC proliferation and invasiveness by mediating translational regulation of WTAP. Studies proved that CCHC-type zinc finger nucleic-acid binding protein (CNBP) recognized METTL3-mediated m^6^A modification of WTAPP1 and recruited RNA stabilizers ELAV like RNA binding protein 1 (Elavl1 or HuR), leading to promoted WTAPP1 stability. Excessive WTAPP1 then recruited translation initiation factor eukaryotic translation initiation factor 3 (EIF3) complex to its protein-coding counterpart WT1-associated protein (WTAP) mRNA, which induced WTAP translation and activates Wnt signaling pathway in a WTAP-initiated manner. More importantly, inhibition of WTAPP1 significantly reduced PDAC growth rates in mouse xenograft tumors models, suggesting a potential therapeutic target for PDAC treatment [122].

Potassium two pore domain channel subfamily K member 15 and WISP2 antisense RNA 1 (KCNK15-AS1) is another lncRNA found to be down-regulated in PC cells and tissues, which has been confirmed to inhibit the migration and invasion of PC cells. Overexpression of KCNK15-AS1 could inhibit cell proliferation, migration and epithelial mesenchymal transformation (EMT) while facilitate cell apoptosis in PC. In terms of mechanism, m^6^A methylation was proved to mediate KCNK15-AS1 downregulation, and m^6^A eraser ALKBH5 could induce m^6^A demethylation of KCNK15-AS1 to mediate KCNK15-AS1 up-regulation. In PC cells, KCNK15-AS1 directly bound to KCNK15 5′UTR and mediated translational regulation to inhibit the expression of KCNK15 to restrain PC cells malignant behaviors. Moreover, KCNK15 also recruited MDM2 proto-oncogene (MDM2) to facilitate RE1 silencing transcription factor (REST) ubiquitination, which further upregulated PTEN to inactivate PTEN/AKT signaling pathway. Taken together, these findings suggest that KCNK15-AS1 may serve as a potential biomarker and therapy target of PC [123].

### Respiratory tract cancers

As is known to all, common cancers of the respiratory system include nasopharyngeal and lung cancer [124]. Thus far, emerging studies have indicated the experience of m^6^A-modified lncRNAs in nasopharyngeal carcinoma, non-small-cell lung cancer, lung adenocarcinoma, lung squamous cell carcinoma and so on.

#### Nasopharyngeal carcinoma

Nasopharyngeal carcinoma (NPC) is an epithelial carcinoma originating from the nasopharyngeal mucosal lining that is often observed at the pharyngeal recess. NPC can be divided into three pathological subtypes, including keratinising squamous, non-keratinising, and basaloid squamous. Among them the non-keratinising subtype accounts most cases in endemic areas (> 95%), which is greatly associated with Epstein-Barr virus (EBV) infection [125]. Recent investigations have shown that m^6^A writer METTL3 displayed an apparent increase while m^6^A writer METTL14 just slightly increased in NPC cell lines [126]. And it was reported that lncRNA ZNFX1 antisense RNA 1 (ZFAS1) could regulate the autophagy level of NPC cells through the miR-100/autophagy related 10 (ATG10) axis and the PI3K/AKT/mTOR pathway to affect tumor progression [127]. Moreover, m^6^A writer METTL3 directly mediated the m^6^A modification of ZFAS1 to reduce the rate of ZFAS1 degradation [127]. Thus, the m^6^A-modified ZFAS1 has significant carcinogenic effects in the occurrence and development of NPC.

Additionally, it was shown that family with sequence similarity 225 member A (FAM225A) was an oncogenic lncRNA that promotes NPC cell proliferation, migration, and invasion via the FAM225A-miR-590-3p/miR-1275/integrin subunit beta 3 (ITGB3)/focal adhesion kinase (FAK)/PI3K/AKT signaling pathway. In terms of mechanism, FAM225A acted as a ceRNA to mediate post-transcriptional regulation and competitively absorbed miR-590-3p and miR-1275 to upregulate ITGB3 expression, which was the target gene of miR-590-3p and miR-1275 [128]. Further, ITGB3 could activate the FAK/PI3K/AKT pathway, which is greatly related to autophagy [129, 130]. In addition, there were 2 RRACU m^6^A sequence motifs in the last exon (at position 2,808 and 5,460) in FAM225A and it was found that m^6^A writer METTL3 directly participate in the m^6^A modification of FAM225A to induce the expression of FAM225A [128]. Taken together, m^6^A-midified FAM225A significantly facilitated NPC progression, indicating a potential biomarker and therapy target of NPC.

#### Lung cancer

With an approximated 2.2 million new cases and 1.79 million new deaths every year, lung cancer is one of the most universally diagnosed cancers and the leading cause of cancer death worldwide [131]. It can be further classified into two broad histological subtypes: non-small-cell lung cancer (NSCLC) and small-cell lung cancer (SCLC). NSCLC is further classified into three subtypes, namely the squamous cell carcinoma, adenocarcinoma, and large-cell carcinoma cancer [132, 133]. For early lung cancer detection, spiral CT is still the method of choice, while liquid biopsies are still under exploration. Till then, using PCR or next-generation sequencing to detect EGFR mutations with ctDNA are the solitary FDA approved plasma tests [131, 134]. Therefore, identifying new biomarkers can be valuable for lung cancer diagnosing.

NSCLC accounts for 85% of all lung cancer cases [132]. It was reported that METTL3/YTHDF3 complex directly induced NSCLC drug resistance and metastasis by regulating the MALAT1-miR-1914-3p-YAP axis. LncRNA MALAT1 functioned as a ceRNA and mediate post-transcriptional regulation that sponged miR-1914-3p to induce NSCLC drug resistance of DDP and metastasis through upregulating YAP, a target of miR-1914-3p. Furthermore, studies identified the RRACH sequence in MALAT1 as the m^6^A modification site and proved that the stability of m^6^A-modified MALAT1 was increased by METTL3/YTHDF3 complex [135]. Additionally, it was shown that lncRNA ABHD11 antisense RNA 1 (ABHD11-AS1) could recruit EZH2 to the promoter region of KLF transcription factor 4 (KLF4) gene, thereby repressing the transcription of KLF4, which might function as a cancer inhibitor to inhibit the NSCLC’s Warburg effect. Moreover, during the transcription of ABHD11‐AS1, METTL3 could enhance ABHD11‐AS1 transcript stability to increase its expression via a m^6^A-dependent way [136]. Taken together, these m^6^A-modified lncRNAs may serve as novel biomarkers for NSCLC detection.

Lung squamous cell carcinoma (LUSC) is the second most prevalent subtype of lung cancer, accounting for around 30% of NSCLC [137]. Further studies have reported that m^6^A- modified lncRNAs might affect patients with LUSC by regulating TME but the precise mechanism remains unclear. Recently, four m^6^A-related lncRNAs, including AC138035.1, AC243919.2, HORMAD2-AS1 and AL122125.1 were identified to be closely related to the prognosis of LUSC based on bioinformatics analysis. In addition, HORMAD2-AS1 was highly expressed, whereas the expression of AC138035.1, AC243919.2, and AL122125.1 was downregulated in the high-risk LUSC compared with the low-risk LUSC [138]. Moreover, it was shown that the expression level of AL122125.1 was correlated with ion channel regulator activity, protein tyrosine kinase binding, histone acetyltransferase binding and ATP transmembrane transporter activity. Additionally, multivariate Cox regression analysis shows that AL122125.1 demonstrated a great prognostic value [139]. To be brief, these lncRNAs are closely related to LUSC and may be novel detection biomarkers and targets for the treatment of LUSC.

Lung adenocarcinoma (LUAD) is the most common histologic subtype of lung cancer, which accounts for over 40% of NSCLC [140]. It was widely proved that the transcription factor E2F transcription factor 1 (E2F1) was a central factor that involved in LUAD cell cycle progression, DNA-damage response, and apoptosis [141]. Recently, lncRNA SVIL antisense RNA 1 (SVIL-AS1), which was located on chromosome 10q13, has been found to be downregulated in LUAD and act as a tumor suppressor in LUAD tumorigenesis in a E2F1-dependent manner. SVIL-AS1 could mediate post-translational regulation on E2F1 by regulating E2F1 protein degradation via controlling E2F1 ubiquitination, which further retarded LUAD progression. It is worth noting that SVIL-AS1 seemly regulated E2F1 at both RNA and protein level rather than the transcriptional level. Additionally, it was reported that m^6^A writer METTL3 directly participated in installing the SVIL-AS1 m^6^A modification and maintaining its stability. In summary, the tumor-suppressive role of the lncRNA SVIL-AS1 was mediated and stabilized by m^6^A writer METTL3 in LUAD [142]. Altogether, these m^6^A-modified lncRNAs may as novel diagnostic biomarkers for LUAD and provide new therapy targets for LUAD.

### Urogenital neoplasms

Urogenital neoplasm is a severe lesion in urogenital system. More than 90% of urogenital neoplasms are malignant, and every urinary and male reproductive organ is likely to develop tumors. In urogenital neoplasms, cancers of the female genital organs including cervical cancer, endometrial cancer, ovarian cancer, uterine cancer, vaginal cancer and vulvar cancer. Cancers of the male genital organs including penile cancer, prostate cancer and testicular cancer. And cancers of the urinary organs including bladder cancer and renal cell carcinoma. Existing evidence has proved the existence of m^6^A-modified lncRNAs in cervical cancer, endometrial cancer, ovarian cancer, bladder cancer, and clear cell renal cell carcinoma.

#### Cervical cancer

Cervical cancer keeps being the fourth most common female malignancy worldwide, which has become a huge health challenge and economic burden, especially in some developing countries [115, 143]. Human papillomavirus (HPV) is the leading cause of cervical cancer, high risk HPV subtypes can cause virtually all cervical cancers and screening and vaccination targeting HPV are efficient disease prevention strategies [143]. For the clinical staging of cervical cancer, PET showed great sensitivity and specificity for detecting involved nodes, but such imaging modalities may be unavailable in some developing countries [143]. Therefore, identifying new biomarkers for cervical cancer detection can be constructive. Up to now, researchers have identified several m^6^A-modified lncRNAs in cervical cancer progression. Y Zhang et al. reported a cervical cancer-related lncRNA called KCNMB2 antisense RNA 1 (KCNMB2-AS1), which was significantly overexpressed in cervical cancer and related to poor outcomes. Inhibition of KCNMB2-AS1 could evidently delay cervical cancer growth in vivo xenograft models. In terms of mechanism, KCNMB2-AS1 could mediate post-transcriptional regulation on miR-130b-5p and miR-4294 by functioning as a ceRNA to abundantly sponge miR-130b-5p and miR-4294, leading to the overexpression of a well-known oncogene IGF2BP3. Meanwhile, IGF2BP3 could also act as a m^6^A reader and bind to KCNMB2-AS1 via recognizing three m^6^A modification domains on KCNMB2-AS1, which prevented the degradation of KCNMB2-AS1. This positive regulatory circuit formed by KCNMB2-AS1 and IGFBP3 significantly induced cervical cancer progression and targeting KCNMB2-AS1 and its related molecules may be a potential detective and therapeutic way for cervical cancer patients [144].

Another m^6^A-modidfied lncRNA FOXD2 adjacent opposite strand RNA 1 (FOXD2-AS1) was also found to be upregulated in cervical cancer tissues and cells, which closely related to the poor prognosis. Functionally, assays showed that FOXD2-AS1 induced the migration and proliferation of cervical cancer cells and silencing of FOXD2-AS1 significantly inhibited cervical cancer growth in vivo. Further studies reported a remarkable m^6^A site in the 3’ UTR of FOXD2-AS1, and METTL3 overexpression upregulated the m^6^A modification level and FOXD2-AS1 stability in cervical cancer cells. Indicating that FOXD2-AS1 was positively regulated by METTL3, METTL3 maintained FOXD2-AS1 overexpression in cervical cancer. Upregulated FOXD2-AS1 then mediated transcriptional regulation on p21 by recruiting a transcriptional silence factor lysine-specific demethylase 1 (LSD1) to p21 promoter and inhibited its transcription, which relieved the tumor suppression mediated by p21, leading to expedited cervical cancer progression. In conclusion, METTL3/FOXD2-AS1 accelerates cervical cancer progression in a m^6^A-dependent manner, suggesting a potential therapeutic target for cervical cancer [145].

#### Endometrial cancer

Endometrial cancer (EC) is one of the most common gynecological malignant diseases, which raised 417,367 new cases and 97,370 new deaths in 2020 [115]. Traditionally, EC can be divided into two types based on clinical, metabolic, and endocrine characteristics. Type1 ECs are estrogen dependent and associated with endometrial hyperplasia, while type2 ECs are estrogen independent and associated with endometrial atrophy [146]. A one-stop clinic with sequential transvaginal ultrasound, endometrial sampling and hysteroscopy can be effective and efficient for EC diagnosing. However, sequential transvaginal ultrasound may be less specific since endometrial thickness fluctuates regularly in healthy reproductive-aged women. Endometrial sampling will be limited by cervical stenosis. Hysteroscopy may raise widely ranged discomfort, from mildly unpleasant to severely painful [147]. Therefore, it’s worthy to explore an efficient and comfortable detection strategy, in which liquid biopsy based m^6^A-modified lncRNAs may have a great potential. Uterine corpus endometrial carcinoma (UCEC) is a common kind of EC. L Shan et al. explored the expression profiles of m^6^A-related lncRNAs of patients with UCEC and identified five m^6^A-modified lncRNAs that might act as novel prognostic markers for UCEC. Via coexpression analysis and pipelining into univariate Cox regression models and risk score models, they screened out five major lncRNAs associated with m^6^A, including AL645568.1, NNT-AS1, RAB11B-AS1, LINC01936 and HM13-IT1. Further researches showed that the risk risk-based model constructed from the five lncRNAs was associated with immune cell infiltration level, which may be a potential accurate prognosticator for UCEC [148].

#### Ovarian cancer

Ovarian cancer is one of the main causes of gynecological cancer-related death worldwide. As the lack of effective detection methods and clinical manifestations in the early stage, 70% of patients are diagnosed in the middle or late stages, which brings more trouble for the treatment [149]. Till now, several risk models have been constructed to independently predict the overall survival and therapeutic value of ovarian cancer based on m^6^A-mdiated lncRNAs. For example, JF Zheng et al. identified ten significant prognostic m^6^A-mediated lncRNAs in OC through univariate Cox regression analysis. According to the least absolute shrinkage and selection operator (LASSO) Cox regression analysis of these ten lncRNAs, they then conducted a prognostic signature containing four m^6^A-mediated lncRNAs (AC010894.3, ACAP2-IT1, CACNA1G-AS1 and UBA6-AS1), which shows great predictive value in ovarian cancer detection [150]. Additionally, Y Song et al. established a nomogram based on the expression level of seven m^6^A-related lncRNAs (AC008669.1, AC010336.1, AC097376.3, AC130710.1, ACAP2-IT1, AL138820.1 and CACNA1G-AS1) to predict survival rate of patients with ovarian cancer, revealing that m^6^A-related lncRNAs may act an important role in ovarian cancer treatment [151].

Epithelial ovarian cancer (EOC) is a common subtype of ovarian cancer. LncRNA RHPN1 antisense RNA 1 (RHPN1-AS1) has been found to be upregulated in EOC tissues and high expression of RHPN1-AS1 is closely associated with poor prognosis in EOC patients. In EOC cells, m^6^A methylation was proved to be part of the cause of RHPN1-AS1 upregulation. M^6^A writer METTL3 mediates m^6^A modification on RHPN1-AS1, which enhances its transcriptional stability, leading to RHPN1-AS1 upregulation. Overexpressed RHPN1-AS1 then acts as a ceRNA and mediates post-transcriptional regulation to sponge miR-596, which increases leucine zipper and EF-hand containing transmembrane protein 1 (LETM1) expression, elevates the levels of p-FAK and p-Akt and further activates the FAK/PI3K/Akt signaling pathway. Taken together, overexpressed RHPN1-AS1 promotes EOC cell proliferation and metastasis through a METTL3/RHPN1-AS1/miR-596/LETM1/FAK/PI3K/Akt axis, indicating that RHPN1-AS1 may provide a promising drug target for EOC treatment [152].

#### Bladder cancer

Bladder cancer (BLCA) is the most common malignancy of the urinary system, accounting for an estimated 500,000 new cases and 200,000 deaths worldwide [153]. For BLCA diagnosing, CT urography and cystoscopy are the most common and effective methods. Two new technologies called blue-light cystoscopy and narrow-band imaging were recently developed to improve the detection of malignant BLCA [154]. As an important sample source for liquid biopsy, urine-based tumor markers may have huge potential in BLCA detection, however, no existing molecular markers have a validated sensitivity high enough to replace cystoscopy [154]. To perfect the cytological or molecular analysis of BLCA, m^6^A-modified lncRNAs may give a new idea. Till now, several m^6^A-related lncRNAs prognostic signatures (m^6^A-RLPS) for predicting the prognosis of BLCA patients have been reported. For example, TM Ma et al. identified 745 m^6^A-related lncRNAs by Pearson correlation analysis, in which 51 prognostic m^6^A-related lncRNAs were screened using univariate Cox regression analysis. Finally, they determined nine m^6^A-related prognostic lncRNAs (AC020911.1, KCNQ1OT1, AC104532.2, AC006160.1, EHMT2-AS1, AC097359.2, AP001469.1, AC007686.3 and AL022322.1) to conduct the m^6^A-RLPS, which was proved to be an independent predictor of BLCA prognosis [155]. Additionally, another study showed a 12 m^6^A-related lncRNAs prognostic score (m^6^A-LRS), including ZNRD1-AS1, SNHG16, SBF2-AS1, RNF217-AS1, RASAL2-AS1, PSMB8-AS1, PINK1-AS, GUSBP11, FAM13A-AS1, C17orf82, C140rf132 and BDNF-AS. High m^6^A-LRS was proved to be associated with tumor-associated biological processes, oncogenic signaling, and tumor hallmarks in BLCA progression. Moreover, an m^6^A-LRS-based nomogram was also conducted, which demonstrates a strong ability to predict overall survival in BLCA patients [156]. In conclusion, these studies provide new approaches for diagnose and treatment response prediction in BLCA.

#### Renal cell carcinoma

Renal cell carcinoma (RCC) is a prevalent malignant tumor of the urinary system with a high mortality and steadily rising morbidity worldwide [115]. Clinically, advanced and metastatic diseases chiefly account for the mortality rates in RCC patients, surgically extirpating all small renal masses might not improve patient outcomes [157]. Therefore, reducing RCC invasiveness is important in clinical treatment. Recently, targeted m^6^A methylation of lncRNAs has been proved to regulate RCC cell proliferation and metastasis, indicating a great potential in RCC treatment. For instance, lncRNA nuclear paraspeckle assembly transcript 1 (NEAT1) expression and m^6^A methylation level is downregulated in RCC tissues, and low NEAT1 expression is associated with poor prognosis for RCC patients. Further assays showed that CRIPSR/dCas13b-METTL3 transfection significantly increases m^6^A methylation level in RCC cells, which increases NEAT1 expression. Meanwhile, hypermethylation of NEAT1 can further inhibit the proliferation and migration of RCC cells, indicating a new target for treatment of RCC [158].

Clear-cell renal cell carcinoma (ccRCC) is the most common subtype of RCC, which contributes to more than 70% of cases [157]. In ccRCC tissues, m^6^A writer METTL14 was downregulated and low level of METTL14 was negatively associated with the prognosis, stage, and ccRCC tumor grade. LncRNA large 60 S subunit nuclear export GTPase 1 (Lnc-LSG1) was identified as a downstream target of METTL14. In ccRCC cells, Lnc-LSG1 could mediate post-translational regulation by directly binding to epithelial splicing regulatory protein 2 (ESRP2) and inhibit its expression through the ubiquitin-proteasome pathway, which further promoted ccRCC metastasis. In this axis, METTL14-mediated m^6^A methylation on Lnc-LSG1 could inhibit the interaction between ESRP2 and Lnc-LSG1 via a YTHDC1-dependent manner. YTHDC1 directly binds to the m^6^A sites on Lnc-LSG1, which competitively inhibits Lnc-LSG1 binding to ESRP2. Taken together, m^6^A-modified Lnc-LSG1 can significantly inhibit ccRCC progression, indicating a novel therapeutic target [159].

### Endocrine tumors

Endocrine tumors consist of malignancies that originated in the endocrine system, such as thyroid, breast, adrenal, and pituitary gland [160]. Thus far, a growing body of studies have demonstrated the involvement of m^6^A-modified lncRNAs in thyroid cancer, and breast cancer. The details were descripted as follows.

#### Thyroid Cancer

Thyroid cancer (TC) is the eighth most frequently diagnosed cancer worldwide with a rising incidence in the past 20 years [161]. With a high negative predictive value and low false negative rate, fine-needle aspiration is widely used cytological diagnosis of TC. However, thyroid nodule fine-needle aspiration cytology may be confounded in diagnostic categories 3 and 4 (indeterminate results), which provides a novel stage to molecular diagnostics. Fairly recently, researchers reported that IGF2BP2/ lncRNA HOXD antisense growth-associated long non-coding RNA (HAGLR) axis was closely related to TC [162]. It was proved that reducing the expression of HAGLR would inhibit papillary thyroid cancer cell proliferation, migration, and invasion, and promoted cell-cycle arrest [163]. Moreover, there was a partial overlapping between a high-confidence m^6^A-modified region and one of the putative binding sites of m^6^A reader IGF2BP2 and HAGLR (chr2: 177,037,923–177,037,952(-)). Thus, it was indicated that this m^6^A site is involved in mediating the regulatory effect of IGF2BP2 on HAGLR. Furthermore, m^6^A reader IGF2BP2 could significantly improve the stability of HAGLR transcripts to upregulate the level of HAGLR by recognizing the m^6^A modification of HAGLR in TC [162]. Taken together, the interaction of HAGLR and m^6^A reader IGF2BP2 is closely related to TC progression, which suggests the potential values of HAGLR in the diagnosis and treatment of TC.

Additionally, both lncRNA MALAT1 and m^6^A reader IGF2BP2 were found to be upregulated in TC tissues. Mechanistically, MALAT1 functioned through a miR-204/IGF2BP2/myelocytomatosis (MYC) axis to confer a stimulatory effect on cell proliferation, migration, and invasion. In this axis, miR-204 could target and inhibit the expression of IGF2BP2, while MALAT1 could mediate post-transcriptional regulation by competitively binding to miR-204 to upregulate IGF2BP2. Upregulated IGFBP2 then recognizes the m^6^A modifications of MYC in TC cells, which induced the expression of MYC and further promotes cell proliferation, migration, and invasion of TC cells [164]. Moreover, researchers also found that METTL3 could form m^6^A modifications on MALAT1, which acted an important role in the subnuclear localization of MALAT1 [165]. Altogether, the m^6^A-modified MALAT1 can be considered as a novel, promising biomarker for TC detection and treatment.

Papillary thyroid carcinoma (PTC) is the most common subtype of TC [166]. It was reported that three m^6^A-related lncRNAs, PSMG3-AS1, BHLHE40-AS1, and AC016747.3, could be used as prognostic markers for patients with PTC based on bioinformatics analysis. The overall survival rate was significantly different with the increasing PSMG3-AS1 and BHLHE40-AS1 expression levels while the disease-free survival rate is significantly different with the increasing expressions of AC016747.3 in PTC. In addition, PSMG3-AS1 and BHLHE40-AS1 were found to correspond to sensitivity of various chemotherapy drugs in PTC and paly an oncogenic role in various cancers [167]. Furthermore, it was proved that based on bioinformatics analysis, 8 m^6^A-lncRNAs, including AC139795.2, TRAM2.AS1, POLR2J4, AC018653.3, DOCK9.DT, GABPB1.AS1, NORAD, and AL022328.2, exhibited a strong prognostic value in PTC treatment [168]. However, the specific mechanism remains to be investigated. In short, these lncRNAs may be considered as new, promising prognostic biomarkers and treatment targets, to a certain degree.

#### Breast cancer

Breast cancer (BC) is still the most common cancer worldwide, which accounts for about 30% of female cancers and has a mortality-to-incidence ratio of 15% [169]. The incidence of breast cancer continues to rise, and it remains the leading cancer-related cause of disease burden for women [170]. Because the extensive characterizing of BC molecular hallmarks, several kinds of biomarkers have been clinically used, including immunohistochemical markers, proliferation marker protein, genomic markers and immunomarkers, which have shown great prospects in BC detection and treatment [171]. As a widely studied regulating factor, m^6^A-modified lncRNAs have shown great correlation with BC cell proliferation, which may serve a lot in future diagnosing and therapy. For instance, it was reported that MIR210 host gene (MIR210HG) was overexpressed in breast cancer tissues and functioned as an oncogenic lncRNA to promote cell proliferation of BC cells. Mechanistically, the functions of MIR210HG were mediated by its encoded miR-210, inhibition of miR-210 significantly prohibited BC invasion ability induced by MIR210HG. Moreover, four potential m^6^A modification sites (chr11:565778, chr11:567257, chr11:567410, chr11:567484) were found in MIR210HG, IGF2BP1 and its co-factor Elavl1 (also known as HuR) mediated recognition of these m^6^A sites could enhance the stability of MIR210HG. Interestingly, IGF2BP1 was also directly activated by MYCN proto-oncogene, bHLH transcription factor (MYCN), which may explain the oncogenic role of MYCN. Taken together, the MYCN/ IGF2BP1/MIR210HG/miR-210 axis may serve as an alternative molecular mechanism of BC progression [172].

**Fig. 6:**
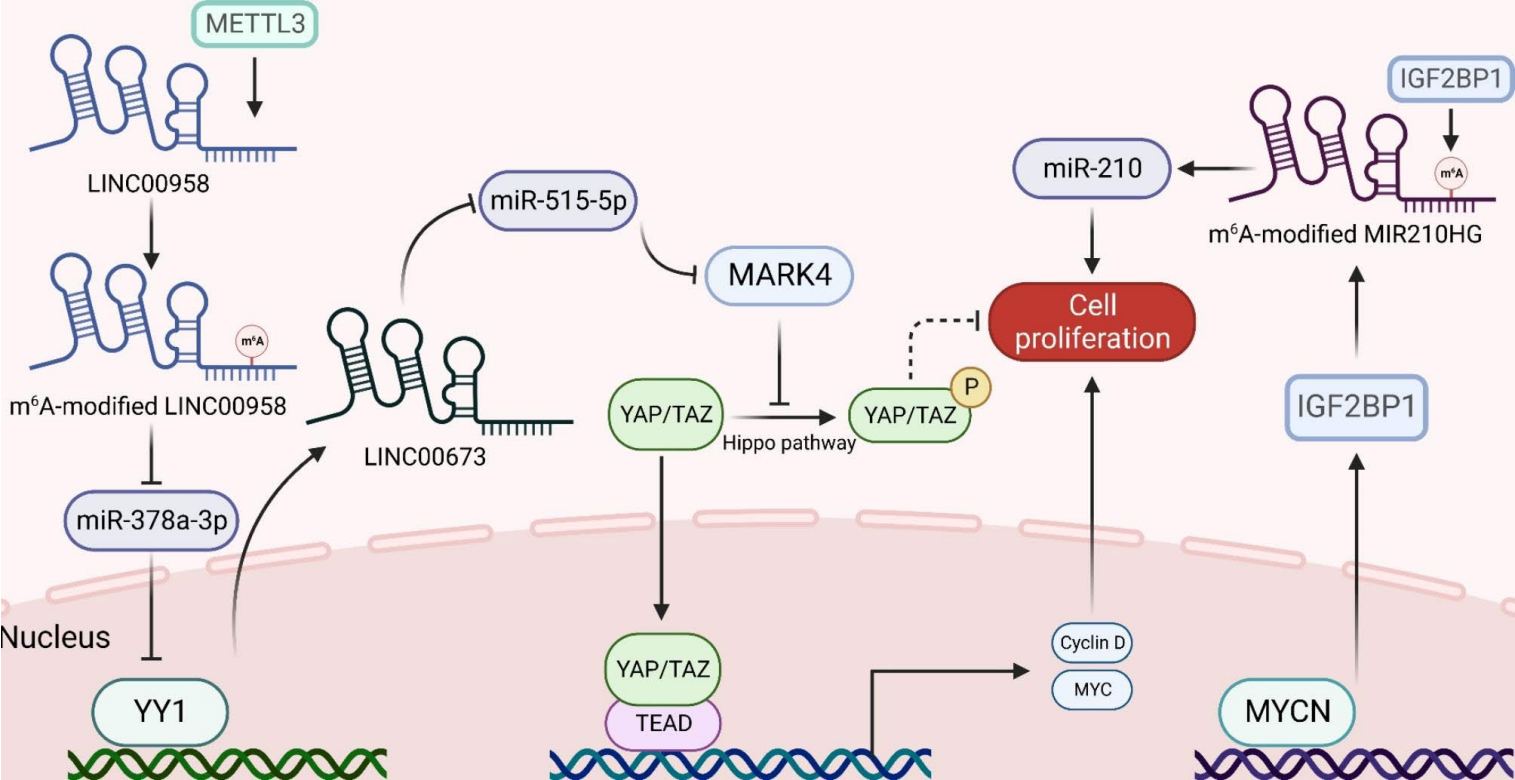
M^6^A-modified lncRNAs in breast cancer. LINC00958 may promote breast cancer cell proliferation via METTL3/LINC00958/miR-378a-3P/YY1/LINC00673/miR-515-5P/MARK4/Hippo axis; MIR210HG promotes breast cancer cell proliferation via MYCN/IGF2BP1/MIR210HG/miR-210 axis

Moreover, lncRNA long intergenic non-protein coding RNA 958 (LINC00958) was also reported to be upregulated in BC tissues and could promote breast cancer tumorigenesis via the miR-378a-3p/YY1 axis. Mechanistically, m^6^A writer METTL3-mediated m^6^A modification promoted the upregulation of lncRNA LINC00958 through improving its stability. Upregulated LINC00958 then mediated post-transcriptional regulation by serving as a ceRNA for miR-378a-3p to positively regulate YY1 [173]. Furthermore, it has been proved that YY1 could promote cell proliferation via the LINC00673/miR-515-5p/microtubule affinity regulating kinase 4 (MARK4)/Hippo signaling pathway [174]. Yes-associated protein 1 (YAP) and transcriptional coactivator with PDZ-binding motif (TAZ) are two major effectors of Hippo pathway. YAP/TAZ cooperate with transcriptional enhanced associate domains (TEADs) to enhance the transcription of growth promoting factors including MYC, cyclin D, which further promotes cell proliferation. Phosphorylation of YAP/TAZ will inhibit the promoting effect, while MARK4 could remove this inhibition [174]. Taken together, m^6^A-modified LINC00958 significantly promotes BC cell proliferation via the miR-378a-3p/YY1 axis, which indicates a potential target for breast cancer detection and treatment [173]. (Fig. 6)

### Hematological malignancies

Hematological malignancy are usually malignant diseases of the blood system. Common blood tumors mainly include various types of leukemia, multiple myeloma, and malignant lymphoma [175]. Thus far, emerging investigations have explored the role of m^6^A modified lncRNAs in diffuse large B cell lymphoma, leukemia and chronic myelocytic leukemia.

#### Diffuse large B cell lymphoma

Diffuse large B cell lymphoma (DLBCL) is a subtype of lymphoma, which accounts for about 30% of all cases of non-Hodgkin’s lymphoma with an estimated 150,000 new cases annually worldwide [176]. Recent studies have demonstrated that lncRNA translation regulatory long non-coding RNA 1 (TRERNA1) was upregulated in DLBCL tissues and was closely associated with the poor prognosis of DLBCL patients [177]. In terms of mechanism, m^6^A eraser ALKBH5-mediated demethylation on TRERNA1 significantly increased the stability and regulation of TRERNA1. Upregulated TRERNA1 then interacted with EZH2, a core subunit of PRC2, and upregulated its expression, which further catalyzed the trimethylation of H3K27me3 to epigenetically silenced the expression of the cyclin-dependent kinases inhibitor p21, leading to enhanced cell proliferation of DLBCL cells [177]. Taken together, TRERNA1 may provide a novel target for the diagnosis and treatment of DLBCL.

#### Leukemia

Leukemia, also known as blood cancer, is a malignant tumor of the hematopoietic system caused by abnormal functioning of the blood-forming tissue in the bone marrow caused by a mutation of DNA in the cells. Chronic myeloid leukemia (CML) is a typical subtype of leukemia accounting for 20% of all adult leukemias with a large number of immature white blood cells accumulating in the bone marrow, which inhibits the normal hematopoiesis of the bone marrow [178, 179]. Till now, assessing the presence of Philadelphia chromosome is still the main diagnose factor. Though some molecular abnormalities and cancer-associated mutations have been found to frequently emerge during the transformation to accelerated phase or blast phase, more mechanism studies are still needed for future clinical applications [179]. Recently, m^6^A modification of lncRNA NEAT1 was found to regulate CML progression, overexpression of NEAT1 could mediate cell viability inhibition and apoptosis promotion of CML cells [180]. The expression level of NEAT1 was found to be significantly downregulated and METTL3-mediated m^6^A modification was proved to be the reason of the aberrant expression of NEAT1. In terms of mechanism, NEAT1 might function via a miR-766-5p/cyclin dependent kinase inhibitor 1 A (CDKN1A) axis. In CML cells, miR-766-5p was upregulated and its target gene CDKN1A was downregulated. Both of the upregulation of miR-766-5p and the downregulation of CDKN1A was proved to reverse the CML inhibition mediated by NEAT1 [180]. Taken together, NEAT1 may act an important role in CML treatment, but more studies are prerequisite to reveal its mechanisms.

Acute myeloid leukemia (AML) is another subtype of leukemia characterized by malignant proliferation of immature bone marrow stem cells in the bone marrow and peripheral blood [181]. Recently, m^6^A-related lncRNAs were found to predict prognosis and indicate immune microenvironment in AML. FM Zhong et al. identified the risk signals related to prognosis of AML patients by using LASSO regression and then constructed a risk model for independent prediction of overall survival in AML patients based on 15 m^6^A-related lncRNAs, including AC025430.1, AFF2-IT1, LINC02593, AC000120.2, AL158163.1, AC048382.1, AL391834, AC008770.3, AL133492.1, AC020916.2 and AJ239328.1. This risk model showed great correlation with clinicopathological factors and immune infiltration levels, which could independently predict AML prognosis, indicating a novel insight in AML treatment [182].

### Bone and soft tissue tumors

Bone and soft tissue tumors are neoplasms formed by proliferation and abnormal differentiation of normal musculoskeletal cells under the long-term action of different initiating and promoting factors. For bone tumors, most of the cases are benign, like osteoid osteoma, osteochondroma and nonossifying fibroma, while malignant bone tumors are mostly osteosarcomas and Ewing sarcoma family of tumors. For soft tissue tumors, the most common of which include lipomas, hemangiomas, giant cell tumors of tendon sheath and rhabdomyosarcoma [183]. Till now, several pilot studies have proved the existence of m^6^A-modified lncRNAs in osteosarcoma and skin cutaneous melanoma.

#### Osteosarcoma

Osteosarcoma is one of the primary bone malignancies that always occur in children, adolescents and young adults. It is an aggressive tumor that occurs from primitive transformed cells of mesenchymal origin that exhibits osteoblastic differentiation and produces malignant osteoid. Till now, because of the lack of efficient early diagnosis, the 5-year-survival rate and prognosis of osteosarcoma patients still remain unsatisfactory [184]. Though osteosarcoma is one of the earliest identified hominin malignancies, there is very few lack specific tumor markers for osteosarcoma. Lactate dehydrogenase and alkaline phosphatase were found to be obviously elevated in some osteosarcoma patients, but more large cohort studies are still needed for further validation [185]. As both of m^6^A methylation and lncRNAs have been already found to regulate osteosarcoma tumorigenesis, m^6^A-modified lncRNAs may bring a novel insight in osteosarcoma diagnosing and treatment. For example, to better diagnose and cure osteosarcoma, researchers have conducted several models based m^6^A-modified lncRNAs to predict the occurrence and prognosis of osteosarcoma. ZG Wu et al. identified 25 m^6^A-meidated lncRNAs that displayed different expression between osteosarcoma tissues and normal tissues, among which the expression levels of ZBTB32 and DEPTOR were downregulated in osteosarcoma tissues and SPAG4 was the opposite, which might be potential remodeling indicators in the tumor microenvironment and prognostic markers in osteosarcoma [186]. Additionally, D Zheng et al. conducted a risk signature based on six m^6^A-modified lncRNAs, including AP003119.2, LINC01816, AL139289.1, AC004812.2, AC005785.1 and AL353804.1, which harbored a promising ability to predict the overall survival of osteosarcoma patients. Further experiments indicated that AC004812.2 might be an effective prognostic biomarker and therapeutic target in osteosarcoma [187].

FOXD2 adjacent opposite strand RNA 1 (FOXD2-AS1) was another m^6^A-modified lncRNA that acted a critical role in osteosarcoma progression. Clinically, osteosarcoma patients with higher FOXD2-AS1 expression had a poorer prognosis compared to those with lower FOXD2-AS1 expression. Further study revealed that m^6^A writer WTAP mediated m^6^A methylation on the 3ʹ-UTR of FOXD2-AS1 in nucleus, which enhanced the stability of FOXD2-AS1 transcripts. FOXD2-AS1 then mediated post-transcriptional regulation by directly binding to forkhead box M1 (FOXM1) mRNA through its m^6^A sites and formed a FOXD2-AS1/m^6^A/FOXM1 complex to intensify FOXM1 mRNA stability [188]. FOXM1 has been proved to function via promoting the expression of c-Jun N-terminal kinase (JNK1) and matrix metalloproteinase 2 (MMP-2) in osteosarcoma. On the one hand, promoted JNK1 could induce the G1/S transition to facilitate cell proliferation. On the other hand, promoted JNK1 could induce the expression of MMP-9, which together with MMP-2 implicated in tumor cell invasion and metastasis by degrading the extracellular matrix [189]. In conclusion, FOXD2-AS1 could promote both cell proliferation and metastasis in osteosarcoma progression.

**Fig. 7:**
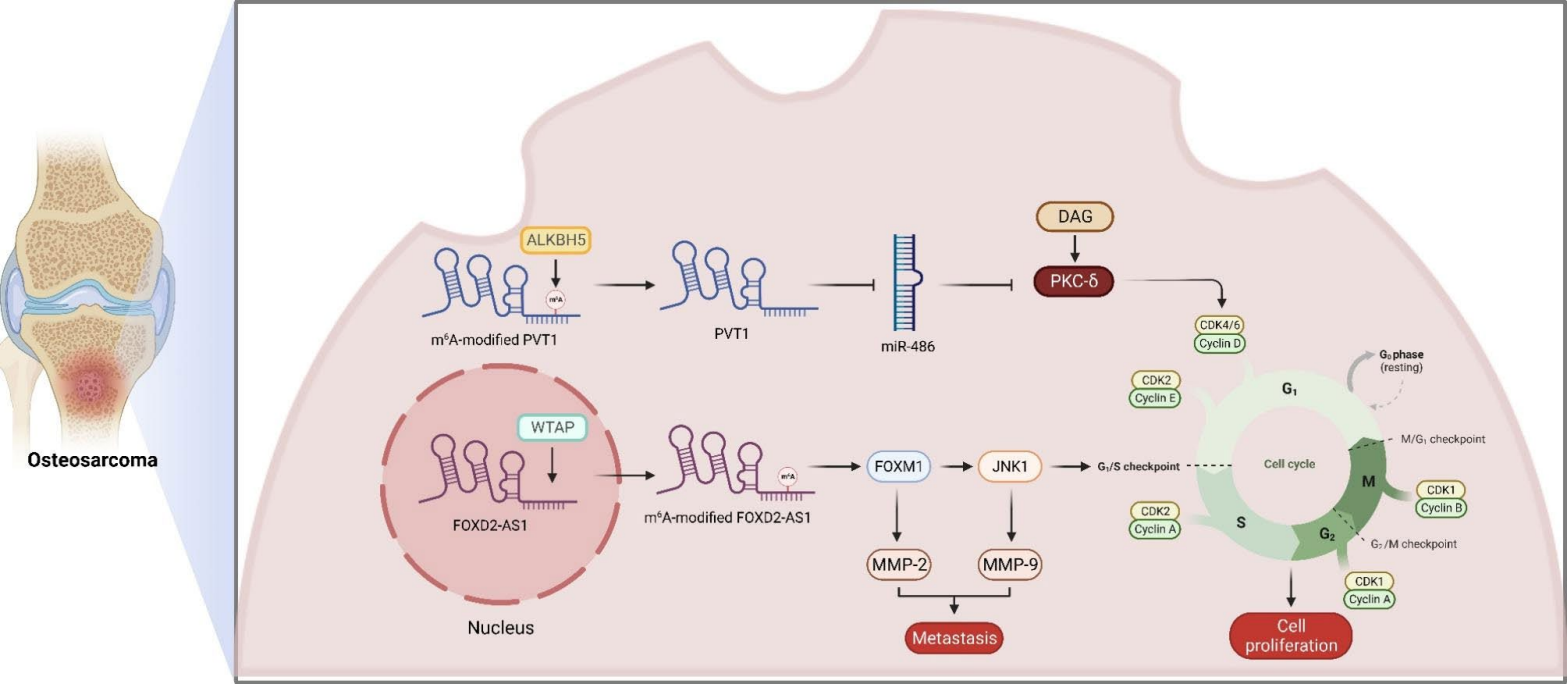
M^6^A-modified lncRNAs in osteosarcoma. FOXD2-AS1 promotes osteosarcoma cell proliferation and metastasis via WTAP/FOXD2-AS1/FOXM1/JNK1/MMP-2/MMP-9 axis; PVT1 promotes osteosarcoma cell proliferation via ALKBH5/PVT1/miR-486/PKC-δ axis

In addition, a well-known oncogenic lncRNA plasmacytoma variant translocation 1 (PVT1) was also found to be upregulated in osteosarcoma tissues and cells, whose expression significantly correlated with clinical stage, tumor size, and prognosis of osteosarcoma patients. Further investigation revealed that the upregulation of PVT1 was due to the interaction between m^6^A eraser ALKBH5 and PVT1. ALKBH5 removed the m^6^A modification on PVT1, which inhibited the binding of reader protein YTHDF2 in PVT1 and further suppresses PVT1 degradation [190]. Upregulated PVT1 then promoted osteosarcoma metastasis via a miR-486/protein kinase C delta S homeolog PKC-δ axis. PVT1 sponges miR-486, which relieved miR-486-mediated PKC-δ signaling pathway silencing [191, 192]. PKC-δ was a key regulator in cell cycle progression that could be activated by diacylglycerol (DAG) and functioned by regulating the expression of cyclins and cyclin-dependent kinases (CDKs), and miR-486 could inhibit CDK4 and CDK6 by silencing PKC-δ [192, 193]. Taken together, this ALKBH5/PVT1/miR-486/PKC-δ axis significantly induced the malignant progression of osteosarcoma, indicating a novel target in osteosarcoma detection and treatment (Fig. 7).

#### Skin cutaneous melanoma

Skin cutaneous melanoma (SKCM) is a common subtype of melanoma whose incidence kept rising more rapidly than any other solid tumor in the past few decades [194]. As a malignant cancer, the median survival and 5 years survival rate of SKCM patients still remain unsatisfactory, which urgently calls effective biomarkers for prognosis and new therapeutic targets. Recently, researchers proved that m^6^A-related lncRNAs may act as potential biomarkers for SKCM Prognosis. SY Huang et al. used univariate Cox regression analysis to test all the 1086 m^6^A-related lncRNAs in SKCM and filtered 130 independent prognostic lncRNAs. Among which LASSO analysis was used to generate an m^6^A-associated lncRNA model (m^6^A-LncM) that contains 24 lncRNAs, including RP11-775D22.3, RP11-383l23.2, RP11-10L12.4, GS1-204l12.4, RP1-149A16.17, XXbac-BPG252P9.10, JPX, RP11-326l11.3, RP11-247L20.4, STK4-AS1, CTD-2291D10.4, RP11-483P21.2, LINC01150, RP11-539L10.2, WAC-AS1, CTD-2647L4.4, CTA-384D8.34, RP11-480l12.7, AC018464.3, LRP4-AS1, RP11-341A22.2, RP11-21l10.2, RP13-379O24.3 and CTD-3064M3.4. All these 24 lncRNAs are significantly associated with overall survival and Stratified survival analysis showed that this model retains its prognostic efficacy in recurrence, radiation therapy and other subgroups, which provides a new insight to improve the prognosis of SKCM [195].

### Nervous system cancers

Nervous system cancers are a heterogeneous group of neoplasms with varied outcomes and management strategies that range from pilocytic astrocytoma, which are very uncommon, noninvasive, and surgically curable, to glioblastoma, the most common malignant brain tumor in adults, which is highly invasive and virtually incurable [196]. Thus far, the important role of m^6^A modified lncRNAs has been implicated in glioma and its subtype glioblastoma.

#### Glioma

Gliomas are the most common and fatal type of malignant tumor of the central nervous system. Basically, gliomas are divided into two kinds: circumscribed gliomas and diffuse gliomas. Circumscribed gliomas are mostly regarded as benign, which can be cured by complete resection. Diffuse gliomas are almost impossible to be cured by resection alone. Clinically, several diagnostic molecular markers have been used to classify diffuse gliomas, like IDH, MGMT and BRAF [197]. Herein, several research showed a potential role of m^6^A-modified lncRNAs as biomarkers in glioma diagnosing and therapy. ZW Tu et al. recently explored the m^6^A-related lncRNAs in glioma by Pearson correlation analysis and then performed univariate Cox regression analysis to screen their prognostic roles in glioma patients. Finally, they constructed a m^6^A-related lncRNA prognostic signature (m^6^A-LPS) with 9 m^6^A-related lncRNAs, including C6orf3, LINC00237, LINC00925, LINC00152, RP4-758J18.2, RP4-773N10.4, LINC00265, LINC00665, RP11-443B20.1. This m^6^A-LPS showed a robust prognostic ability in the stratification analysis, indicating a novel insight in glioma treatment [198].

Glioblastoma (GBM) is the most common and devastating primary malignant subtype of glioma. Even with surgical resection and the use of highly aggressive therapies, recurrence is inevitable, and the median survival of GBM patients is only 1 year [199]. To improve the outcomes of GBM patients, new diagnostic and therapeutic scheme can be urgently needed. Recently, lncRNA cancer susceptibility 9 (CASC9) was found to be significantly upregulated in GBM tissues and its ectopic high expression was associated with poor survival, which acted as an independent prognostic factor for GBM patients [200]. Further mechanism assays showed that m^6^A reader IGF2BP2 directly bound to the m^6^A site of CASC9 and enhanced its stability, which resulted in the upregulation of CASC9 in GBM. Overexpressed CASC9 then cooperated with IGF2BP2 to form a CASC9/IGF2BP2 complex, which further increased the Hexokinase 2 (HK2) mRNA stability, leading to accelerated aerobic glycolysis [200]. In a word, this CASC9/IGF2BP2/HK2 axis significantly promoted the aerobic glycolysis of GBM, indicating a potential role in GBM detection and treatment.

### Other malignancies

In this last part, we summarized the m^6^A-modified lncRNAs in squamous cell carcinomas (SCCs). SCCs are a group of cancers that originate from squamous and non-squamous epithelial tissues. Based on the location where they appear, SCCs can be classified into different types, like skin, head and neck, esophagus, lung and cervix [201]. Till now, m^6^A-modified lncRNAs have been proved to exist in head and neck SCCs (HNSCCs) and its subtypes: oral SCCs and laryngeal SCCs.

HNSCCs are the seventh most common type of cancers worldwide that arise in the head and neck, which often develop from the mucosal epithelium in the oral cavity, pharynx and larynx [202]. Recently, ZY Feng et al. identified 29 m^6^A-modified lncRNAs that are perspective and highly correlated with a positive prognosis. Among which LASSO regression analysis was conducted and four important m^6^A-modified lncRNAs were identified, including GRHL3-AS1, AL121845.4, AC116914.2 and AL513190.1. A risk signature was conducted from these four lncRNAs and the risk signature score could predict survival rates and discriminate prognosis of HNSCC patients to a certain extent. Meanwhile, the risk score also correlated to clinicopathological factors, clusters and immune-scores in HNSCC, indicating a remarkable value for predicting HNSCC patient prognosis [203].

In addition, lncRNA LNCAROD was found to be upregulated in HNSCC tissues and correlates to advanced T stage and shortened overall survival. Functional studies showed that overexpression of LNCAROD could promote cell proliferation and mobility in vitro, tumorigenicity in vivo, while inhibition of LNCAROD exerted opposite effects. Mechanistically, m^6^A methylation mediated by METTL3 and METTL14 enhanced the stability of LNCAROD in HNSCC cells, resulting LNCAROD upregulation. Overexpressed LNCAROD mainly distributed in nucleus and acts as a bridge between Y-box binding protein 1 (YBX1) and heat shock protein family A (Hsp70) member 1 A (HSPA1A), which facilitated YBX1-HSPA1A interaction to protect YBX1 from proteasomal degradation, leading to promoted malignant development of HNSCC. These findings indicated that LNCAROD is an oncogenic lncRNA in HNSCC and dysregulation of m^6^A modification could facilitate the oncogenic function, suggesting that m^6^A-modified LNCAROD might serve as a potential detective biomarker and therapeutic target of HNSCC [204].

Oral SCCs (OSCC) is the most common subtype of HNSCC that arises in oral mucosa, smoking and drinking have been proved to be the main risk factors of OSCC [205]. Most OSCC patients are detected at the late-stage and the overall prognosis of OSCC is poor, which urgently calls effective diagnostic methods and therapeutic schedules [115]. Recently, Q Yang et al. identified 16 m^6^A-modified lncRNAs that were associated with OSCC patients’ survival prognosis, including AC079684.2, AC092115.4, LINC01644, LINC01410, AL355574.1, AC091271.1, AC006449.5, LINC00630, ALMS1-lT1, LINC00992, AC099850.4, AC005288.1, AC107027.3, JPX, LINC01775 and PTOV1-AS1. These lncRNAs were also found to correlate with several cancer metastasis genes, including Src, Myc and c-Myb. A reliable m^6^A-related lncRNA prognostic model for OSCC patients was further demonstrated based on these lncRNAs, suggesting a great value in OSCC treatment [206].

Moreover, JL Li et al. found that METTL14 and lncRNA MALAT1 were both upregulated in OSCC tissues, silencing of METTL14 significant inhibited the viability and cell proliferation of OSCC cells. Further study showed that METTL14 mediated m^6^A methylation on MALAT1, which facilitated MALAT1 expression. Overexpressed MALAT1 then targeted to miR-224-5p and decreased its expression, which further enhanced lysine demethylase 2 A (KDM2A) transcription, leading to promoted OSCC tumorigenesis. Taken together, this METTL14/MALAT1/miR-224-5p/ KDM2A axis significantly facilitated OSCC progression, indicating a novel diagnostic and therapeutic target [207].

Laryngeal SCCs (LSCC) is the second common subtype of HNSCC and the most common pathological subtype of laryngeal carcinoma, with 184,615 new cases and 99,840 new deaths in 2020 [115, 208]. LncRNA KCNQ1 overlapping transcript 1 (KCNQ1OT1) was found to be low m^6^A methylated and upregulated in LSCC tissues. Depletion of KCNQ1OT1 could significantly inhibit cell proliferation, migration and invasion of LSCC Cells, while overexpression of KCNQ1OT1 enhanced LSCC growth and metastasis. Mechanistically, ALKBH5 mediated m^6^A methylation on KCNQ1OT1 and YTHDF2 then recognized the m^6^A sites, which promoted KCNQ1OT1 expression. Upregulated KCNQ1OT1 then directly bound to homeobox A9 (HOXA9) and induced its expression to further trigger the development of LSCC. This m^6^A/ KCNQ1OT1/ HOXA9 axis may provide a new insight in LSCC diagnosis and treatment [209].

## Diagnostic and therapeutic applications of m^6^A-modified lncRNAs in cancer treatment

Given to the significant roles of m^6^A-modified lncRNAs in cancer metastasis, cell proliferation, angiogenesis, glycolysis and drug resistance, m^6^A-modified lncRNA may have a great potential to serve as a new diagnostic biomarker and therapeutic target in cancer treatment.

As we mentioned before, several m^6^A-modified lncRNAs have been proved to be differently expressed in cancer tissues, for example, upregulated LINC00958 in HCC tissues [118], downregulated NEAT1 in RCC tissues [158], and upregulated FOXD2-AS1 in osteosarcoma tissues [188]. More important, the expression level of these m^6^A-modified lncRNAs were proved to be able to indicate the survival rate of patients, suggesting a great potential in monitoring the cancer progression. What’s more, with the developing of m^6^A RIP-seq and other second-generation sequencing technologies, detecting the expression level of specific m^6^A-modified lncRNAs can be convenient and fast, which makes the idea of using m^6^A-modified lncRNAs as biomarkers technically feasible [210]. Till now, there are already several research have put this idea into practice, for instance, L Shan et al. conducted a risk model consist of five m^6^A-modified lncRNAs to predict the immune cell infiltration level in UCEC tissues [148], D Zheng et al. conducted a risk signature based on six m^6^A-modified lncRNAs to predict the overall survival of osteosarcoma patients [187]. Collectively, these findings provide a new insight for the application of m^6^A-modified lncRNAs in cancer diagnosis and prognosis.

In cancer treatment, m^6^A-modified lncRNAs and their associated proteins may also provide new therapeutic targets. As noted earlier, changing the expression level of specific m^6^A-modified lncRNA can significantly alter tumor progression. For example, silencing of FOXD2-AS1 significantly inhibited cervical cancer growth [145], indicating a potential application of FOXD2-AS1 in cervical cancer treatment. There are also researches have proposed some new therapeutic methods, say, XL Zu et al. developed a novel PLGA-based nanoplatform encapsulating si-LINC00958 for HCC systemic administration, which showed satisfactory antitumor efficacy in HCC PDX models [118]. Chuandong Lang et al. targeted PCAT6 with antisense oligonucleotides (ASO), which showed great therapeutic potential against the bone metastasis of prostate cancer [89]. Moreover, as almost all of these m^6^A-modified lncRNAs are differently expressed in a m^6^A-related manner, regulating the level of specific m^6^A writer, reader or eraser can also be a great manner in cancer treatment. For instance, in ESCC cells, FTO mediated the m^6^A demethylation of LINC00022 and promoted LINC00022 expression in an YTHDF2-dependent manner, upregulated LINC00022 then promoted the cell proliferation and tumor growth of ESCC [116]. In this axis, inhibiting the expression level of m^6^A eraser FTO and m^6^A reader YTHDF2 or inducing the expression level of m^6^A writer like METTL3 may be a feasible treatment method of ESCC. In addition, as mentioned before, P. Cody He et al. recently identified EJCs as m^6^A suppressors, which could inhibit the m^6^A methylation of exon junction-proximal RNA within coding sequences [4]. This epoch-making discovery significantly supplements the current understanding of m^6^A methylation progression, which may provide some new regulating methods of m^6^A level in lncRNAs. Taken together, these findings afford new opportunities for the utilization of m^6^A-modified lncRNAs as therapeutic targets in cancer treatment.

## Conclusion and future perspective

In this review, we summarized the physiological functions of m^6^A regulators, biogenesis and biological functions of m^6^A target lncRNAs and, most importantly, the identified m^6^A-modified lncRNAs in cancers of eight organ systems and their roles in tumorigenesis. As for m^6^A regulators, writers can mediate m^6^A methylations on targeted RNAs while erasers can eliminate these modifications. Readers then recognize the m^6^A changes on RNAs and affect their splicing, transport, degradation, translation and other biological processes. For lncRNAs, RNA Pol II and general transcription factors transcribed lncRNAs have been proved to act an important role in epigenetic modification, transcriptional regulation, post-transcriptional regulation, translational regulation and post-translational regulation. Furthermore, dysregulation of m^6^A-modified lncRNAs and their regulatory proteins have been widely found in numerous kinds of cancers, which indicates a new insight in cancer diagnosis and treatment.

To clinically use m^6^A-modified lncRNAs as diagnostic biomarkers, there are still several questions to be addressed. First, one m^6^A-modified lncRNA can exist in several different cancers. For example, LNCAROD was found to be upregulated both in HCC and HNSCC tissues, and the upregulation of LNCAROD showed malignant tumorigenesis and poor prognosis in both two cancers [98, 204]. It is important to exclude the interference of other tumors in early diagnosis of certain tumor by adopting m^6^A-modified lncRNAs as potential biomarkers.

Second, as several m^6^A-related lncRNAs signatures and risk models have been conducted to predict the prognosis of cancers, the biological function of the lncRNAs in these signatures and models still remain to be elucidated. For example, Zheng et al. conducted a prognostic signature in OC detection based on four m^6^A-modified lncRNAs AC010894.3, ACAP2-IT1, CACNA1G-AS1 and UBA6-AS1, but the specific functions and mechanisms of these lncRNAs have not been annotated [150]. As mentioned above, m^6^A-modified lncRNAs can function in various aspects to regulate cancer progression, including metastasis, cell proliferation, angiogenesis, glycolysis and drug resistance. Despite a set of breakthroughs have been made in this field, more rigid studies are still needed to further unveil the specific clinicopathologic significance and underlying mechanism of m^6^A-modified lncRNAs in cancers. Functional and mechanical studies on these lncRNAs may help to better understand the progression and of m^6^A methylation in cancers and further provide more potential detective biomarkers and therapeutic targets.

Third, as an essential constituent part of tumorgenesis, unusual angiogenesis can be widely found in most of solid cancers, which has already been an important indicator of cancer progression. Furthermore, research has also revealed that lots of lncRNAs exert a vital role in cancer angiogenesis. For example, a small regulatory peptide of STAT3 (ASRPS) was found to inhibit triple-negative breast cancer angiogenesis, and lncRNA cervical cancer associated DHX9 suppressive transcript (lnc-CCDST) was found to regulate angiogenesis of cervical cancer [211, 212]. But for m^6^A-modified lncRNAs, only one lncRNA, namely lnc-CTHCC, was reported to promote angiogenesis in HCC [96]. The interaction between m^6^A-modified lncRNAs and cancer angiogenesis may be worthy of in-depth investigations.

Last, it should be noted that most of previous studies on m6A-modified lncRNAs in cancers are based on in vitro assay and bioinformatic analysis, while animal studies or multi-center, large clinical cohort are still lacking. Therefore, more in vivo experiments or clinical studies with large sample size are still necessary for further validation.

## Data Availability

The data used to support this study are included within the article.

## Abbreviations

1/2-sbsRNAs: Half-STAU1-binding site RNAs
ABHD11-AS1: ABHD11 antisense RNA 1
AKT3: AKT serine/threonine kinase 3
ALKBH5: AlkB homolog 5
AML: Acute myeloid leukemia
ASO: Antisense oligonucleotides
ASRPS: A small regulatory peptide of STAT3
ATG10: Autophagy related 10
AURKB: Aurora kinase B
BC: Breast cancer
BC200: Brain cytoplasmic RNA 1
BLCA: Bladder cancer
BOP1: BOP1 ribosomal biogenesis factor
CASC9: Cancer susceptibility 9
CBLL1: Cbl proto-oncogene like 1
CCAT2: Colon cancer associated transcript 2
CCNL1: Cyclin L1
ccRCC: Clear-cell renal cell carcinoma
CCR4: NOT transcription complex subunit 1
CDK: Cyclin-dependent kinase
CDK19: Cyclin dependent kinase 19
CDKN1A: Cyclin dependent kinase inhibitor 1 A
CML: Chronic myeloid leukemia
CNBP: CCHC-type zinc finger nucleic-acid binding protein
CNOT1: ceRNA:Competitive endogenous RNA
CRC: Colorectal cancer
CT: Cancer-testis
ctRNA: Circulating tumour DNA
CUL4B: Cullin 4B
DAG: Diacylglycerol
DDX21: DEAD-box helicase 21
DGCR8: DGCR8 microprocessor complex subunit
DLBCL: Diffuse large B cell lymphoma
DICER1-AS1: Antisense RNA1 of DICER1
dsRNA: Double-stranded RNA
E2F1: E2F transcription factor 1
EBV: Epstein-Barr virus
EC: Endometrial cancer
EEC: Endometrioid endometrial carcinoma
eIF3: Eukaryotic translation initiation factor 3
eIF4G: Eukaryotic translation initiation factor 4G
EMT: Epithelial mesenchymal transformation
EOC: Epithelial ovarian cancer
ERK: Extracellular regulated MAP kinase
ESCC: Esophageal squamous cell carcinoma
ESCs: Embryonic stem cells
ESRP2: Epithelial splicing regulatory protein 2
EZH2: Enhancers of zeste 2 polycomb repressive complex 2
f^6^A: N6-formyladenosine
FAK: Focal adhesion kinase
FAM225A: Family with sequence similarity 225 member A
Fbxo45: F-box protein 45
FENDDR: FOXF1 adjacent non-coding developmental regulatory RNA
FOXD2-AS1: FOXD2 adjacent opposite strand RNA 1
FOXM1: Forkhead box M1
FTO: FTO alpha-ketoglutarate dependent dioxygenase
G3BP1: G3BP stress granule assembly factor 1
GAS5: Growth arrest-specific 5
GBM: Glioblastoma
H3K27me3: Histone H3 lysine 27 trimethylation
H3K56: Hst3 histone H3 K56
HAGLR: HOXD antisense growth-associated long non-coding RNA
HCC: Hepatocellular carcinoma
HDAC1: Histone deacetylase 1
HDGF: Hepatoma-derived growth factor
HK2: Hexokinase 2
hm^5^C: 5-hydroxymethylcytosine
hm^6^A: N6-hydroxymethyladenosine
hnRNP: Heterogeneous nuclear ribonucleoprotein
HNSCC: Head and neck squamous cell carcinoma
HOTAIR: HOX transcript antisense RNA
HOX: Homeobox
HOXA9: Homeobox A9
HPV: Human papillomavirus
Hsp70: Heat shock protein family A
HSPA1A: Hsp70 member 1 A
IGF1R: Insulin like growth factor 1 receptor
IGF2BPs: Insulin-like growth factor 2 mRNA-binding proteins
IRS1: Insulin receptor substrate 1
ITGB3: Integrin subunit beta 3
JNK1: C-Jun N-terminal kinase
KCNK15-AS1: Potassium two pore domain channel subfamily K member 15 and WISP2 antisense RNA 1
KCNMB2-AS1: KCNMB2 antisense RNA 1
KCNQ1OT1: KCNQ1 overlapping transcript 1
KDM2A: Lysine demethylase 2 A
KLF4: KLF transcription factor 4
LASSO: Least absolute shrinkage and selection operator
LDHA: Lactate dehydrogenase A
LETM1: Leucine zipper and EF-hand containing transmembrane protein 1
LINC00958: Long intergenic non-protein coding RNA 958
LINC01320: Long intergenic non-protein coding RNA 1320
LNCAROD: LncRNA activating regulator of DKK1
lnc-CCDST: LncRNA cervical cancer associated DHX9 suppressive transcript
lncIRS1: LncRNA insulin receptor substrate 1
Lnc-LSG1: LncRNA large 60 S subunit nuclear export GTPase 1
lncPRESS1: LncRNA p53 regulated and ESC associated 1
lncRNA: Long non-coding RNA
LSD1: Lysine specific demethylase 1
LUAD: Lung adenocarcinoma
LUSC: Lung squamous cell carcinoma
LSCC: Laryngeal squamous cell carcinoma
m^1^A: N1-methyladenosine
m^5^C: 5-methylcytosine
m^6^A: N6-methyladenosine
m^6^A-LncM: m6A-associated lncRNA model
m^6^A-LPS: M6A-related lncRNA prognostic signature
m^6^A-LRS: M^6^A-related lncRNAs prognostic score
m^6^A-RLPS: M^6^A-related lncRNAs prognostic signatures
MALAT1: Metastasis-associated lung adenocarcinoma transcript 1
MAPK: Mitogen activated kinase-like protein
MDM2: MDM2 proto-oncogene
METTL3: Methyltransferase-like 3
METTL14: Methyltransferase-like 14
METTL16: Methyltransferase-like 16
MIR210HG: MIR210 host gene
miRNA: MicroRNA
MM: Multiple myeloma
MMP-2: Matrix metalloproteinase 2
mRNA: Message RNA
MTC: Methyltransferase complex
MYC: Myelocytomatosis
MYCN: MYCN proto-oncogene, bHLH transcription factor
NEAT1: Nuclear-enriched abundant transcript 1
NPC: Nasopharyngeal carcinoma
NS: Nuclear speckle
NSCLC: Non-small-cell lung cancer
nt: Nucleotides
OSCC: Oral squamous cell carcinoma
PC: Pancreatic cancer
PCAT6: Prostate cancer associated transcript 6
PDAC: Pancreatic ductal adenocarcinoma
PGK1: Phosphoglycerate kinase 1
PI3K/AKT: Phosphatidylinositol 3-kinase/AKT serine/threonine kinase
PIK3CA: Phosphatidylinositol-4,5-bisphosphate 3-kinase catalytic subunit alpha
PIK3CD: Phosphatidylinositol-4,5-bisphosphate 3-kinase catalytic subunit delta
PIC: Pre-initiation complex
PKC-δ: Protein kinase C delta S homeolog
PKM2: Pyruvate kinase M2
Pol I: RNA polymerase I
PRC2: Polycomb Repressive Complex 2
PTBP1: Polypyrimidine tract binding protein 1
PTC: Papillary thyroid carcinoma
PVT1: Plasmacytoma variant translocation 1
RBM15/15B: RNA-binding motif protein 15/15B
RCC: Renal cell carcinoma
REST: RE1 silencing transcription factor
RGG: RRM and Arg-Gly-Gly
RHPN1-AS1: RHPN1 antisense RNA 1
RNPII: RNA Polymerase II
RP11: RP11-138 J23.1
RUNX2: RUNX family transcription factor 2
SCC: Squamous cell carcinoma
SCLC: Small-cell lung cancer
SHARP: SMART/HDAC1-associated repressor protein
Siah1: Siah E3 ubiquitin protein ligase 1
SIRT6: Sirtuin 6
SKCM: Skin cutaneous melanoma
SLC2A1: Solute carrier family 2 member 1
SLERT: SnoRNA-ended lncRNA that enhances pre-rRNA transcription
SMART: Silencing mediator for retinoid and thyroid hormone receptor
snoRNA: Small nucleolar RNA
SOX4: SRY-related HMG box transcription factor 4
SP1: Sp1 transcription factor
SPHK1: Sphingosine kinase 1
SRSF3: Serine and arginine rich splicing factor 3
SRSF10: Serine and arginine rich splicing factor 10
ssDNA: Single-stranded DNA
STAU1: Staufen 1
SNHG1: Small nucleolar RNA host gene 1
SVIL-AS1: SVIL antisense RNA 1
TAFs: TBP-associated factors
TBP: TATA-binding protein
TC: Thyroid cancer
TF: Transcription factor
THAP7-AS1: THAP7 antisense RNA 1
TRERNA1: Translation regulatory long non-coding RNA 1
UCEC: Uterine corpus endometrial carcinoma
VIRMA: Vir like m6A methyltransferase associated
WHO: World Health Organization
WTAP: WT1 associated protein
WTAPP1: Wilms tumor 1 associated protein pseudogene 1
XCI: X-chromosome inactivation
XIST: X inactive specific transcript
YAP: Yes-associated protein
YAP1: Yes1 associated transcriptional regulator
YTH: YT521-B homology
YTHDC1: YTH domain containing 1
YTHDC2: YTH domain containing 2
YTHDF1: YTH domain family protein 1
YTHDF2: YTH domain family protein 2
YTHDF3: YTH domain family protein 3
YY1: YIN‑YANG‑1
YBX1: Y-box binding protein 1
ZC3H13: Zinc finger CCCH-type containing 13
Zeb1: Zinc finger E-box binding homeobox 1
ZFAS1: ZNFX1 antisense RNA 1
